# Antifungal and Therapeutic Evaluation of l- and d‑Enantiomers of a Plant Defensin-Inspired Peptide against *Sporothrix schenckii* and *Sporothrix brasiliensis*

**DOI:** 10.1021/acsomega.6c04139

**Published:** 2026-06-04

**Authors:** Jeniffer Cristina Serpa da Rosa Santos, Thomas Zacarone Afonso Guimarães, Érica de Oliveira Mello, André de Oliveira Carvalho, Valdirene Moreira Gomes, Gabriel Bonan Taveira

**Affiliations:** Laboratório de Fisiologia e Bioquímica de Microrganismos, Centro de Biociências e Biotecnologia, 28109 Universidade Estadual do Norte Fluminense Darcy Ribeiro, Campos dos Goytacazes, RJ 28013-602, Brazil

## Abstract

Sporotrichosis is an emerging subcutaneous mycosis and an important public and veterinary health concern in Brazil. The ongoing expansion of feline-associated transmission, particularly involving *Sporothrix brasiliensis*, has reinforced the need for new antifungal strategies. In this study, we evaluated the antifungal potential of the bioinspired peptide L-*Ca*Def2.1_G27‑K44_ (L-CDF-GK) and its enantiomer D-*Ca*Def2.1_G27‑K44_ (D-CDF-GK), derived from a defensin identified in *Capsicum annuum* fruits. Antifungal activity was assessed through *in vitro* and *in vivo* assays, together with the investigation of possible mechanisms of action. The analyses included fungal growth inhibition, cytoplasmic membrane permeabilization, oxidative stress induction, mitochondrial depolarization, and erythrocyte cytotoxicity. *In vivo*, *Galleria mellonella* larvae were used to evaluate therapeutic potential by survival analysis, melanization quantification, and hemocyte density determination. Both peptides exhibited significant dose-dependent antifungal activity against *Sporothrix schenckii* and *S. brasiliensis*. The L-isoform completely inhibited the growth of both species at 100 μM, whereas the D-isoform inhibited 90% of *S. schenckii* growth and 70% of *S. brasiliensis* growth. IC_50_ analysis and functional assays performed with *S. schenckii* showed that both peptides induced membrane permeabilization, increased reactive oxygen species production, and loss of mitochondrial membrane potential. In the *G. mellonella* infection model, lethal dose determination indicated greater virulence of *S. brasiliensis*. Both peptides showed low toxicity and significantly improved the survival of larvae infected with *S. schenckii*, with minimal activation of cellular and humoral immune responses. Together, these findings support the potential of CDF-GK-derived peptides as promising candidates for the development of new antifungal strategies against sporotrichosis.

## Graphical abstract

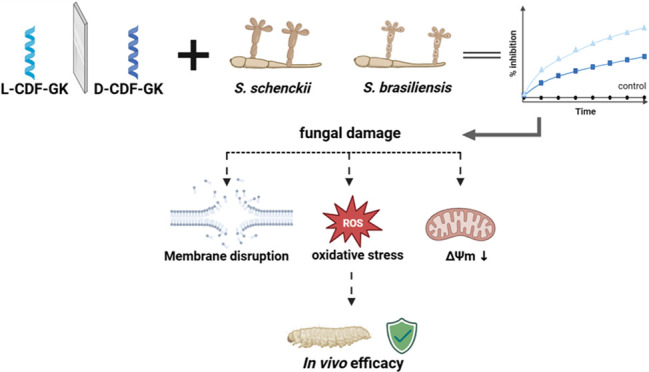

## Introduction

Antimicrobial resistance (AMR) is one of the main global public-health threats. Recent estimates indicate that bacterial AMR directly caused 1.14 million deaths and was associated with 4.71 million deaths worldwide in 2021. Projections further suggest that if current trends persist, AMR-attributable mortality could reach 1.91 million deaths per year by 2050, with 8.22 million deaths associated with resistant bacterial infections globally.^1^ Although these estimates refer specifically to bacterial AMR, they highlight the broader magnitude of antimicrobial resistance as a global health challenge. Within this context, fungal infections represent an increasingly relevant, yet still neglected, component of the AMR crisis. The WHO fungal priority pathogens list, published in 2022, highlights the global health relevance of fungal pathogens and the ongoing limitations in their diagnosis and treatment.^2^ Recent reviews indicate that fungal diseases affect billions of people and cause more than 2 million deaths annually worldwide.^3^ When only severe fungal infections are considered, over 150 million people are affected, leading to more than 1 million deaths each year.^4,5^ In Brazil, recent reviews continue to indicate that more than 3.8 million individuals may be affected by serious fungal infections, particularly people living with HIV, transplant recipients, patients with malignancies, asthmatics, and populations exposed to endemic pathogenic fungi, although this estimate still largely derives from the original national burden analysis.^6,7^

Among fungal diseases of growing concern, sporotrichosis deserves particular attention because of its expanding geographic range and marked zoonotic transmission in Brazil, where *Sporothrix brasiliensis*-driven outbreaks have become a major public health concern.^8,9^ Sporotrichosis is a subcutaneous implantation mycosis caused by thermodimorphic fungi of the genus *Sporothrix*. Historically, the disease has been associated with traumatic inoculation of fungal propagules from soil, plant material, or decaying organic matter, which led to its classical designation as “gardener’s disease”.^10–12^ Molecular taxonomy has clarified that isolates historically grouped under *Sporothrix schenckii* represent distinct pathogenic species within the genus *Sporothrix*, including *S. schenckii*, *S. brasiliensis*, *Sporothrix globosa*, and *Sporothrix luriei*, which are recognized as members of the main pathogenic clade.^13,14^ In Brazil, *S. brasiliensis* has emerged as the predominant and most clinically relevant species, strongly associated with the ongoing zoonotic epidemic. Its epidemiological success is closely linked to feline transmission, since infected cats often harbor a high fungal burden in lesions and act as major sources of infection for other animals and humans.^15–17^ In addition, among pathogenic fungi of the genus *Sporothrix*, *S. brasiliensis* is widely recognized as one of the most virulent species and has been associated with severe, atypical, and extracutaneous clinical presentations, particularly in vulnerable hosts.^9,18,19^

The management of sporotrichosis remains challenging. Itraconazole (ITZ) is still the first-line treatment for most cutaneous forms, whereas amphotericin B is reserved for severe, disseminated, or extracutaneous disease, including cases requiring systemic treatment during pregnancy.^20–22^ However, a reduced therapeutic response in *S. brasiliensis* has been increasingly recognized as a relevant clinical concern.^23,24^ This reduced response has been associated with virulence-related traits such as melanization and efflux pump overexpression.^25,26^ In particular, melanin enhances fungal persistence within the host by protecting against oxidative stress and phagocytosis and has also been linked to decreased susceptibility to ITZ.^11,27,28^ Together, these factors, combined with the still limited availability of structured surveillance and species-oriented therapeutic strategies, reinforce the need for new antifungal approaches against sporotrichosis.^7,22,29^

Against this therapeutic background, antimicrobial peptides (AMPs) have emerged as promising candidates for antifungal therapy. AMPs are small innate immune molecules produced by a wide range of organisms and are typically characterized by cationic charge and amphipathicity, properties that favor selective interaction with microbial membranes.^30,31^ Recent studies and reviews also indicate that rational design can improve key physicochemical features of antifungal peptides, including charge, hydrophobicity, structural stability, and host-cell selectivity, thereby enhancing their therapeutic potential.^32,33^ In addition to membrane disruption, antifungal peptides may induce oxidative stress and interfere with essential intracellular processes, while often retaining broad antifungal activity, limited host toxicity, and a relatively low propensity to induce resistance.^33,34^ Within this framework, our research group previously identified the defensin *Ca*Def2.1 from *Capsicum annuum* and used it as the basis for the rational design of *Ca*Def2.1_G27‑K44_, hereafter referred to as L-CDF-GK, a synthetic peptide corresponding to residues G27–K44 of the native molecule, with the primary amino acid sequence GLTRLRRILFRLLLWRTK, and optimized to increase charge, hydrophobicity, and membrane-active α-helical propensity.^35–37^ Its enantiomeric counterpart, D-*Ca*Def2.1_G27‑K44_, hereafter referred to as D-CDF-GK, has the same primary amino acid sequence, but is composed of D-amino acids. D-CDF-GK was considered as a strategy to improve resistance to proteolytic degradation while preserving biological activity, since D-amino acid substitution is widely recognized as an effective approach to enhance peptide stability.^30,38^ In previous studies, L-CDF-GK showed potent fungicidal activity, a multifactorial mechanism of action, and low toxicity in experimental models.^36,37^ Therefore, comparing L-CDF-GK and D-CDF-GK may provide relevant insight into the development of new antifungal strategies against *S. schenckii* and *S. brasiliensis*.

In light of the therapeutic challenges posed by sporotrichosis and the promising antifungal properties of bioinspired peptides, this study evaluated the antifungal potential of L-CDF-GK and D-CDF-GK against *Sporothrix* spp. by using complementary *in vitro* and *in vivo* approaches. We examined their inhibitory activity against *S. schenckii* and *S. brasiliensis* as well as their effects on plasma membrane permeabilization, reactive oxygen species production, and mitochondrial membrane potential. In addition, we assessed peptide toxicity in *Galleria mellonella* and evaluated the *in vivo* therapeutic efficacy of both peptides in the *S. schenckii* infection model. Together, these analyses provide an integrated view of the antifungal activity, mechanism of action, and safety profile of CDF-GK-derived peptides and support their potential as candidates for the development of new therapeutic strategies against sporotrichosis.

## Materials and Methods

### Strains and Culture Conditions

*Sporothrix schenckii* ATCC 32286 and a clinical isolate of *S. brasiliensis* (189), kindly provided by Prof. Dr. Susana Johann from the Department of Microbiology at the Federal University of Minas Gerais (UFMG), were used in this study. The fungi were maintained by cryopreservation in the yeast phase at −20 °C in the fungal collection of LFBM until reactivation for the experiments. For reactivation, cryotubes were thawed and the isolates were subcultured on 2% Sabouraud dextrose agar and incubated at 25 °C for 5 days to allow microbial growth in the conidial form.

### *In vitro* Antifungal Susceptibility Assays

Antifungal susceptibility was evaluated by broth microdilution using yeast cells. Cells from the different *Sporothrix* strains (1 × 10^4^ cells mL^–1^) were incubated in brain heart infusion (BHI) broth containing different peptide concentrations (200 to 1.56 μM) in a final volume of 100 μL. Assays were performed in 96-well cell culture plates at 37 °C for 72 h. Optical density was measured at 620 nm every 24 h for 3 days. Untreated fungal cells were used as growth controls, and ITZ (25 μM) was used as the positive antifungal control. The 50% inhibitory concentration (IC_50_) was defined as the peptide concentration (μM) that inhibited 50% of yeast growth and was estimated by nonlinear regression analysis.^36^ To determine whether the complete growth inhibition observed with L-CDF-GK was associated with a fungistatic or fungicidal effect, a viability assay was performed. *S. schenckii* and *S. brasiliensis* cells were treated with 100 μM L-CDF-GK under the same conditions used in the broth microdilution assay. After 72 h of incubation, the contents of wells containing untreated control cells or cells treated with L-CDF-GK were collected, centrifuged, washed with peptide-free BHI broth to minimize peptide carryover, and plated onto peptide-free BHI agar using a Drigalski loop. The plates were incubated at 37 °C for 48 h, and fungal growth was assessed by visual inspection of colony formation. Untreated control cells were considered viable when colony growth was observed under the same conditions. Colony formation after peptide removal was interpreted as a fungistatic effect, whereas the absence of colony growth was interpreted as a fungicidal effect. The assay was performed in triplicate

### Membrane Permeabilization Assay

Membrane permeabilization in *S. brasiliensis* and *S. schenckii* was evaluated by fluorescence microscopy using the Sytox Green probe. Cells were incubated with the IC_50_ of D-CDF-GK or L-CDF-GK for 72 h. Control and treated cells were then incubated with 0.2 μM Sytox Green for 10 min at 37 °C and analyzed by differential interference contrast (DIC) microscopy using an optical microscope (Axioplan.A2, Zeiss) equipped with a fluorescence filter set for fluorescein detection (excitation, 450–490 nm; emission, 500 nm).^39^ For each fluorescence assay, all images were acquired using the same microscopy settings, including magnification, fluorescence intensity, exposure time, and camera parameters, allowing qualitative comparison between untreated and peptide-treated cells within the same experiment.

### Effects of the Peptides on ROS Induction

To evaluate whether the peptides induced oxidative stress in *Sporothrix* cells, intracellular reactive oxygen species were measured in *S. schenckii* and *S. brasiliensis* using the fluorescent probe 2′,7′-dichlorofluorescein diacetate (H_2_DCFDA). Cells were incubated with the IC_50_ of D-CDF-GK or L-CDF-GK for 72 h. After incubation, control and treated cells were exposed to 20 μM H_2_DCFDA for 30 min at 37 °C and analyzed by DIC microscopy using an optical microscope (Axioplan.A2, Zeiss) equipped with a fluorescence filter set for fluorescein detection (excitation, 450–490 nm; emission, 500 nm).^40^

### Evaluation of Mitochondrial Membrane Potential (Δψm)

Mitochondrial membrane potential (Δψm) was evaluated only in *S. schenckii* using 5,5′,6,6′-tetrachloro-1,1′,3,3′-tetraethylbenzimidazolocarbocyanine iodide (JC-1). Cells were incubated with the IC_50_ of D-CDF-GK or L-CDF-GK for 72 h. After incubation, cell suspensions were centrifuged at 2500 rpm for 15 min, washed once with 500 μL PBS (10 mM NaH_2_PO_4_, 0.15 M NaCl, pH 7.4), and resuspended in 50 μL PBS. The cells were then incubated with 2 μM JC-1 for 30 min at 30 °C. Negative control cells, incubated in the absence of D-CDF-GK or L-CDF-GK, were processed under the same conditions. Cells were analyzed by DIC microscopy using an optical microscope equipped with a fluorescence filter for fluorescein detection (excitation, 450–490 nm; emission, 530 and 590 nm).^41^

### Hemolytic Activity

The hemolytic potential of D-CDF-GK and L-CDF-GK was assessed using defibrinated sheep red blood cells (sRBCs), following the procedure of Oren and Shai^42^ with modifications. Before the assay, sRBCs were washed in 0.15 M NaCl by centrifugation at 2400*g* for 10 min and then resuspended in the same solution. Peptide samples were prepared in saline, and 50 μL of each concentration (200, 100, 50, or 25 μM) was mixed with 50 μL of the erythrocyte suspension to obtain a final cell concentration of 1% (v/v). The mixtures were incubated for 1 h at 37 °C and then centrifuged at 2400*g* for 10 min. Subsequently, the supernatants were transferred to a 96-well plate, and hemoglobin release was quantified by measuring absorbance at 405 nm. Erythrocytes treated with 1% Triton X-100 were used as the positive control (C^+^), whereas erythrocytes incubated in saline alone served as the negative control (C^–^). Each condition was analyzed in three independent experiments. Hemolytic activity was calculated by considering the Triton X-100 group as 100% hemolysis, according to the equation: % hemolytic activity = 100 × (peptide_ABS405_ – C_ABS405_^–^)/(C_ABS405_^+^ – C_ABS405_^–^). Data are presented as the mean of three independent experiments performed in triplicate. Dose–response curves were generated, and statistical significance (***p < 0.001) relative to the Triton X-100 group was determined using Tukey’s test.

### Larval Maintenance and Experimental Handling

*G. mellonella* larvae were obtained from colonies maintained at LFBM/CBB/UENF and reared on the diet described by Jorjão et al.^43^ Sixth-instar larvae weighing 0.25 ± 0.3 g and showing no visible signs of melanization were selected for the experiments and distributed into groups of 10 in Petri dishes. Before inoculation, the prolegs were disinfected with 70% ethanol. Yeast and peptide suspensions were administered using 10 μL insulin syringes (Uniqmed; 8 mm × 0.30 mm needle, 5/16″ × 30G). Larval mortality was determined based on body melanization and lack of movement in response to gentle stimulation with forceps.

### Larval Toxicity and Hemocyte Response

The *in vivo* toxicity of D-CDF-GK and L-CDF-GK was assessed in *G. mellonella* according to Mylonakis et al.,^44^ with modifications. Groups of 15 last-instar larvae weighing 250–300 mg were used for each treatment. Each larva received 10 μL of a 500 μM peptide solution injected into the hemocoel through the last proleg using an insulin syringe. Three control groups were included to monitor basal larval viability: one injected with PBS, one injected with DMSO, and one subjected only to needle puncture. After treatment, larvae were maintained in Petri dishes at 37 °C, and mortality was recorded every 24 h for 7 days. Larvae were considered dead when they failed to respond to touch. Survival data were plotted as Kaplan–Meier curves, and differences among groups were analyzed using the log-rank Mantel–Cox and Breslow–Wilcoxon tests in GraphPad software (version 8.0.2).

To further examine peptide toxicity, hemocyte density was determined at 3, 6, and 24 h after injection. Hemolymph was collected from larvae treated with 10 μL of PBS, DMSO, 500 μM D-CDF-GK, or 500 μM L-CDF-GK. Before collection, larvae were surface-disinfected with 70% ethanol. Individual hemolymph samples (10 μL) were obtained after puncturing the last proleg with an insulin needle and transferred to microcentrifuge tubes. Samples were immediately diluted 1:10 in ice-cold insect physiological saline (IPS; 150 mM NaCl, 5 mM KCl, 100 mM Tris/HCl, 10 mM EDTA, 30 mM sodium citrate, pH 6.9), centrifuged at 800*g* for 5 min at 4 °C, and the pellets were resuspended in cold IPS. Hemocytes were then counted using a Neubauer chamber (Laboroptik).^45^

### *In vivo* Infection and Therapeutic Assays in *G. mellonella*

To establish the *in vivo* infection model, the lethal fungal dose of the different *Sporothrix* strains was determined in *G. mellonella*. For this purpose, 10 μL of serially diluted fungal suspensions prepared in PBS (30 mM), corresponding to final inocula ranging from 10^5^ to 10^7^ CFU/larva, were injected into the hemocoel through the last right proleg using insulin syringes. Noninoculated larvae were included as controls. After inoculation, larvae were incubated at 37 °C in the dark and monitored individually every 24 h for 15 days. Larvae that failed to respond to touch were considered dead. For each condition, 20 larvae were used, and each experiment was repeated at least twice in triplicate. Survival curves were generated as percentages, and differences among groups were analyzed by the Kaplan–Meier method using the log-rank Mantel–Cox and Breslow–Wilcoxon tests in GraphPad software (GraphPad Software, Inc., California, CA, USA). Based on the lethal dose determination, the therapeutic efficacy of the peptides was evaluated in larvae infected with *S. schenckii*. Each larva was inoculated with 10^6^ CFU/larva through the last left proleg, and separate groups were subsequently treated with D-CDF-GK or L-CDF-GK at 150 or 300 μM, ITZ at 150 μM, or PBS. Treatment solutions were injected into a different proleg from that used for fungal inoculation. After treatment, larvae were maintained in Petri dishes at 37 °C, and mortality was recorded every 24 h for 15 days. The entire experiment was performed twice in triplicate.^37,45^

### Hemocyte Density and Melanization Analysis

To assess the cellular immune response, groups of five *G. mellonella* larvae were inoculated with 10^6^ CFU/larva of *S. schenckii* and incubated at 37 °C. Hemocyte density was determined 3 and 24 h after fungal inoculation and treatment with 300 μM D-CDF-GK or L-CDF-GK. Untreated larvae and larvae injected only with PBS were used as negative controls. Hemolymph extraction and hemocyte quantification were performed as described above.

Melanization was evaluated in parallel using groups of five larvae inoculated with 10^6^ CFU/larva of *S. schenckii* and incubated at 37 °C. 24 h after fungal inoculation and treatment with 300 μM D-CDF-GK or L-CDF-GK, hemolymph was collected from each larva as described above. Untreated larvae and PBS-injected larvae were included as negative controls. Hemolymph samples were diluted 1:10 in insect physiological saline (IPS), centrifuged at 4500*g* for 5 min at 4 °C, and the optical density of the supernatant was measured at 405 nm using a spectrophotometer.^45^

## Results

### Antifungal Activity against *S. brasiliensis* and *S. schenckii*

Initially, the antifungal potential of the synthetic peptides L-CDF-GK and D-CDF-GK was evaluated against *S. schenckii* and *S. brasiliensis*. In both species, a significant concentration-dependent reduction in fungal growth was observed after 72 h of incubation at 37 °C (Figure 1). D-CDF-GK inhibited approximately 90% of *S. schenckii* growth and about 70% of *S. brasiliensis* growth at 100 μM (Figure 1B). In contrast, L-CDF-GK completely inhibited the growth of both species at the same concentration (Figure 1A). Nonlinear regression analysis was used to estimate the IC_50_ values (Figure 1C). D-CDF-GK showed greater activity against *S. schenckii* (IC_50_ = 9.1 μM) than against *S. brasiliensis* (IC_50_ = 26.9 μM). In contrast, L-CDF-GK displayed similar IC_50_ values for both species, with 34.9 μM for *S. schenckii* and 32.6 μM for *S. brasiliensis*. As a positive control, ITZ reduced the growth of both species (Figure 1), confirming the susceptibility of the strains to the reference antifungal.

**Figure 1:**
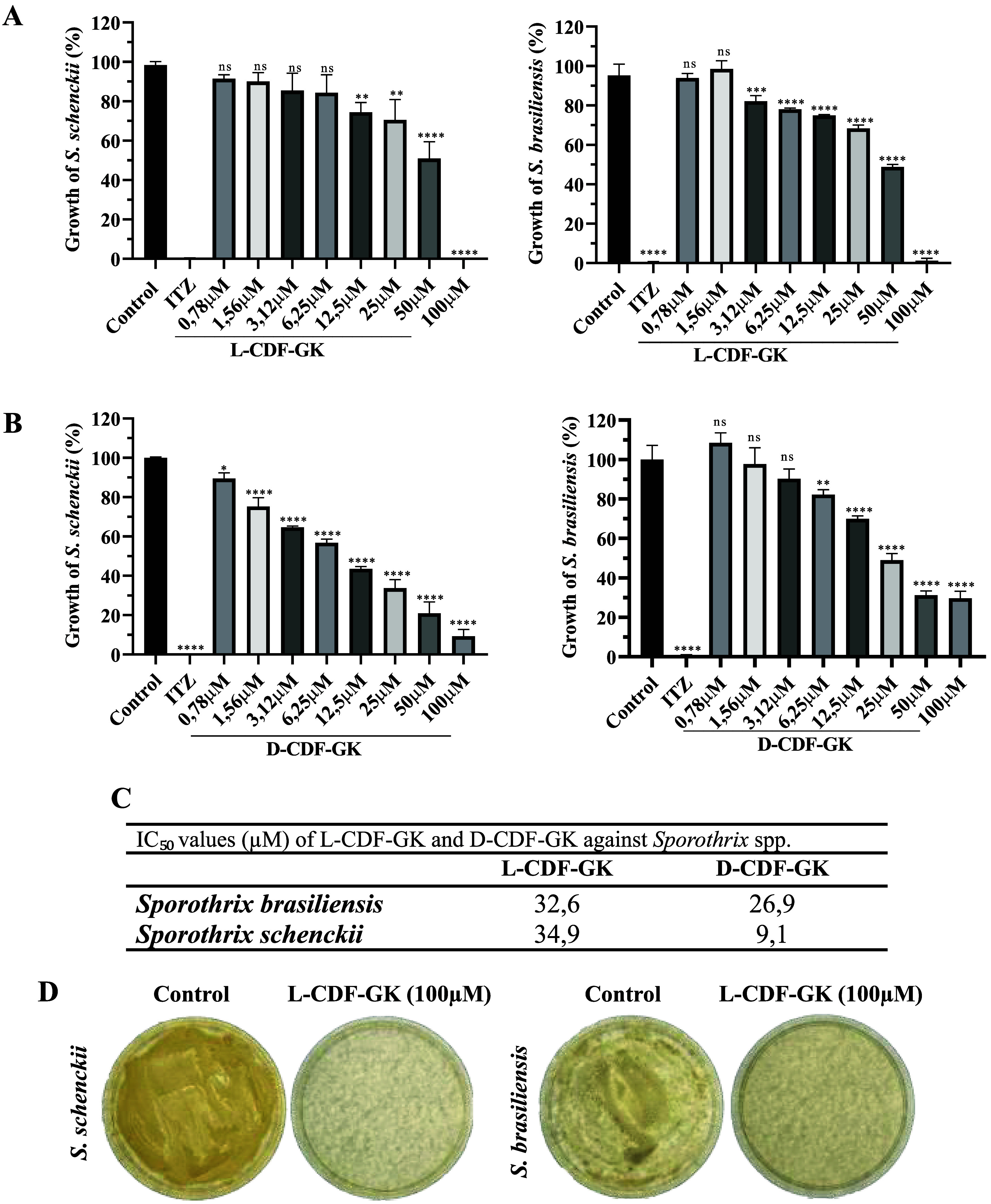
Effect of (A) L-CDF-GK and (B) D-CDF-GK on the growth of *S. schenckii* and *S. brasiliensis* at concentrations ranging from 200 to 1.56 μM after 72 h of incubation. Values represent the mean ± SD of triplicates. Asterisks indicate significant differences between treatments and controls: *p < 0.05, **p < 0.01, ***p < 0.001, and ****p < 0.0001. ITZ, itraconazole (25 μM). (C) Shows the corresponding IC_50_ values obtained by nonlinear regression analysis. IC_50_ was defined as the peptide concentration that inhibited 50% of fungal growth, as estimated by nonlinear regression analysis. (D) Viability assay after treatment with 100 μM L-CDF-GK for 72 h. Treated and untreated cells were plated onto peptide-free BHI agar and incubated at 37 °C for 72 h. The absence of colony growth after peptide treatment indicates loss of fungal viability and supports a fungicidal effect under the tested conditions.

Because L-CDF-GK completely inhibited the growth of both *S. schenckii* and *S. brasiliensis* at 100 μM, we further evaluated whether this effect was associated with loss of fungal viability. After treatment with 100 μM L-CDF-GK for 72 h, cells were plated onto peptide-free BHI agar to assess viability by colony formation. No colony growth was observed for either *S. schenckii* or *S. brasiliensis*, indicating that L-CDF-GK rendered fungal cells nonviable and exerted a fungicidal effect under these experimental conditions (Figure 1D).

### Membrane Permeabilization in *S. schenckii* and *S. brasiliensis*

The Sytox Green assay showed that both peptides induced membrane permeabilization in *S. schenckii* and *S. brasiliensis* (Figure 2A,B). No fluorescence was detected in control cells incubated only with Sytox Green, confirming the plasma membrane integrity. In contrast, cells treated with L-CDF-GK and D-CDF-GK exhibited intense fluorescent staining, accompanied by a marked reduction in the number of viable cells. In *S. schenckii*, morphological alterations were more pronounced, with strongly stained cells and diffuse fluorescence throughout the field, suggesting greater membrane damage than that observed in *S. brasiliensis*. Although both species showed membrane permeabilization, the fluorescent signal was visually stronger in *S. schenckii*, indicating a higher susceptibility of this species to the membrane-disrupting effects of the peptides.

**Figure 2:**
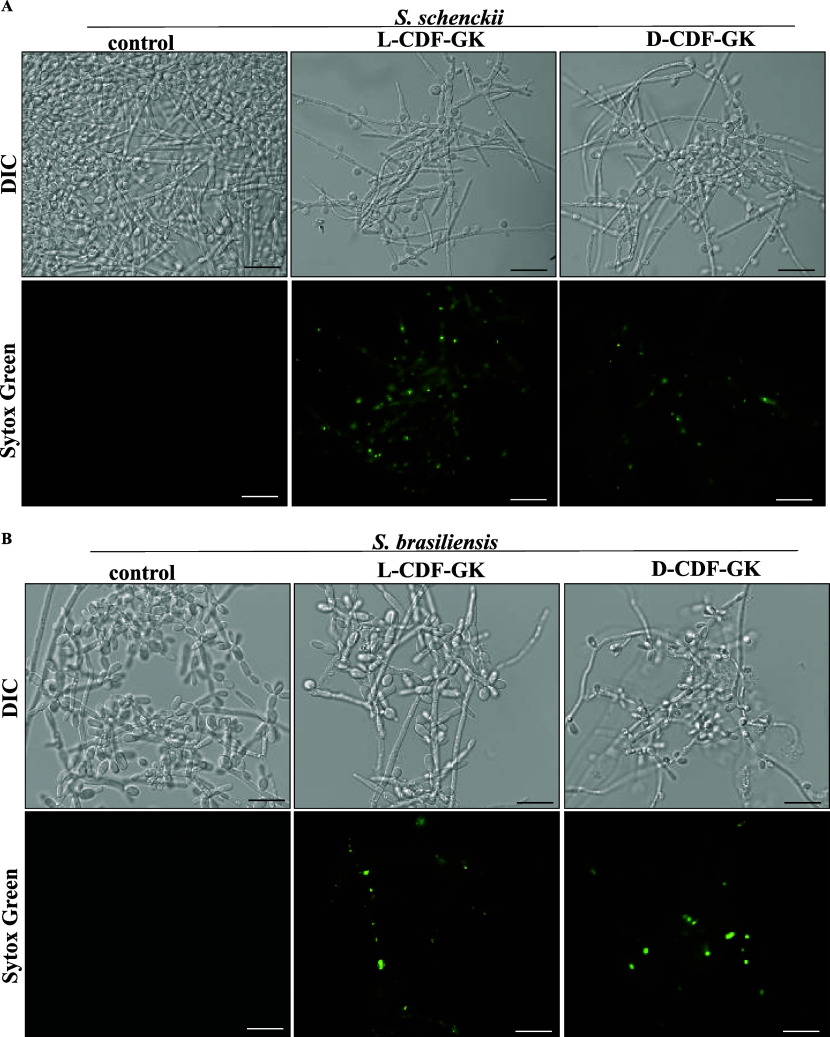
Fluorescence microscopy images of (A) *S. schenckii* and (B) *S. brasiliensis* after membrane permeabilization assay using the Sytox Green probe. Cells were treated with the IC_50_ of each peptide, incubated at 37 °C for 72 h, and then assessed for membrane permeabilization. Control cells were incubated with Sytox Green only. Scale bars = 20 μm.

### Endogenous ROS Production

Reactive oxygen species production was assessed by using the H_2_DCFDA probe (Figure 3). No fluorescence was detected in control cells incubated with only the probe, indicating basal ROS levels. In contrast, treatment with L-CDF-GK or D-CDF-GK resulted in a marked increase in fluorescence in both *S. schenckii* and *S. brasiliensis*, demonstrating the induction of oxidative stress in both species. Consistent with the membrane permeabilization assay, *S. schenckii* exhibited a stronger fluorescent signal than *S. brasiliensis*, suggesting a greater ROS accumulation in this species following peptide treatment. Taken together with the Sytox Green data, these results indicate that the antifungal activity of L-CDF-GK and D-CDF-GK involves both plasma membrane disruption and increased ROS production, with a more pronounced effect in *S. schenckii*.

**Figure 3:**
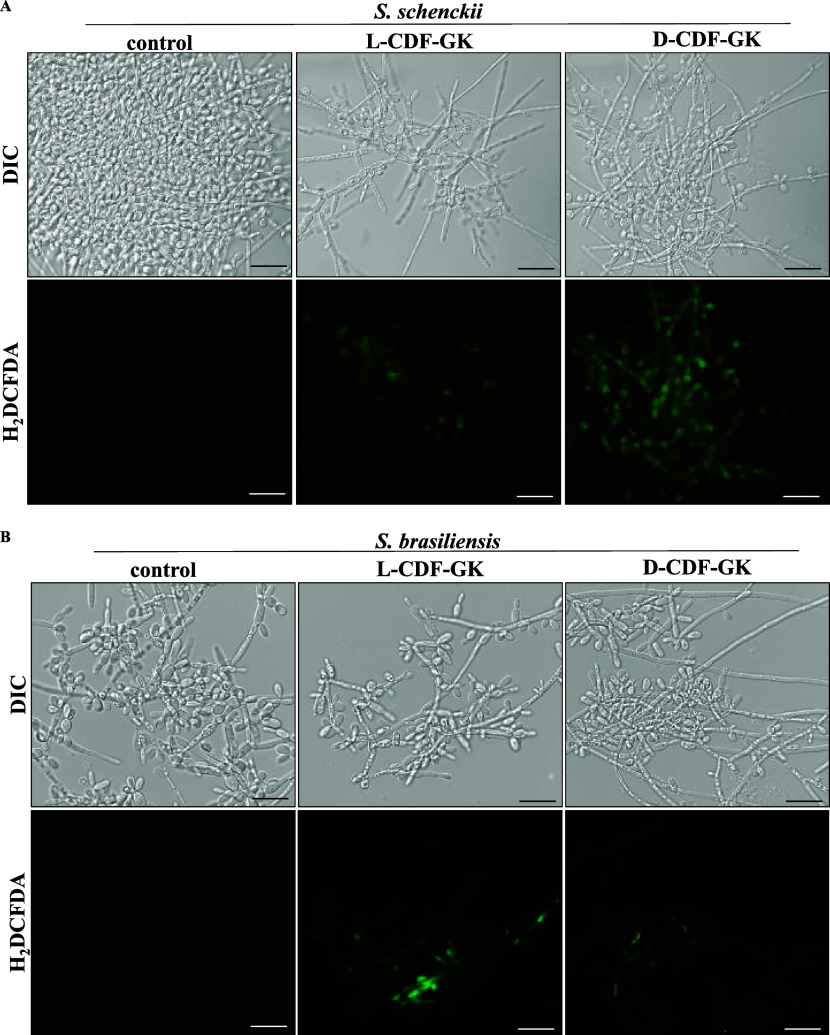
Fluorescence microscopy images of (A) *S. schenckii* and (B) *S. brasiliensis* after reactive oxygen species (ROS) induction assay using the H_2_DCFDA probe. Cells were treated with the IC_50_ of each peptide for 72 h and then evaluated for oxidative stress. Control cells were incubated with H_2_DCFDA only. Scale bars = 20 μm.

### Mitochondrial Membrane Potential (Δψm) in *S. schenckii*

Based on its greater susceptibility to peptide treatment in the preceding assays, particularly regarding membrane permeabilization and ROS accumulation, *S. schenckii* was selected for further mechanistic evaluation of mitochondrial membrane depolarization. The effect of the peptides on the Δψm of *S. schenckii* was evaluated by fluorescence microscopy using the JC-1 probe (Figure 4). In control cells, red fluorescence predominated, corresponding to the formation of J-aggregates in the mitochondrial matrix, which is characteristic of mitochondria with preserved Δψm. After treatment with L-CDF-GK or D-CDF-GK, a marked reduction in red fluorescence and an increase in green fluorescence associated with the monomeric form of JC-1 were observed. This shift in fluorescence pattern is consistent with loss of mitochondrial membrane potential in *S. schenckii* cells after peptide treatment. These observations suggest that mitochondrial depolarization occurs as part of the cellular alterations associated with peptide exposure.

**Figure 4:**
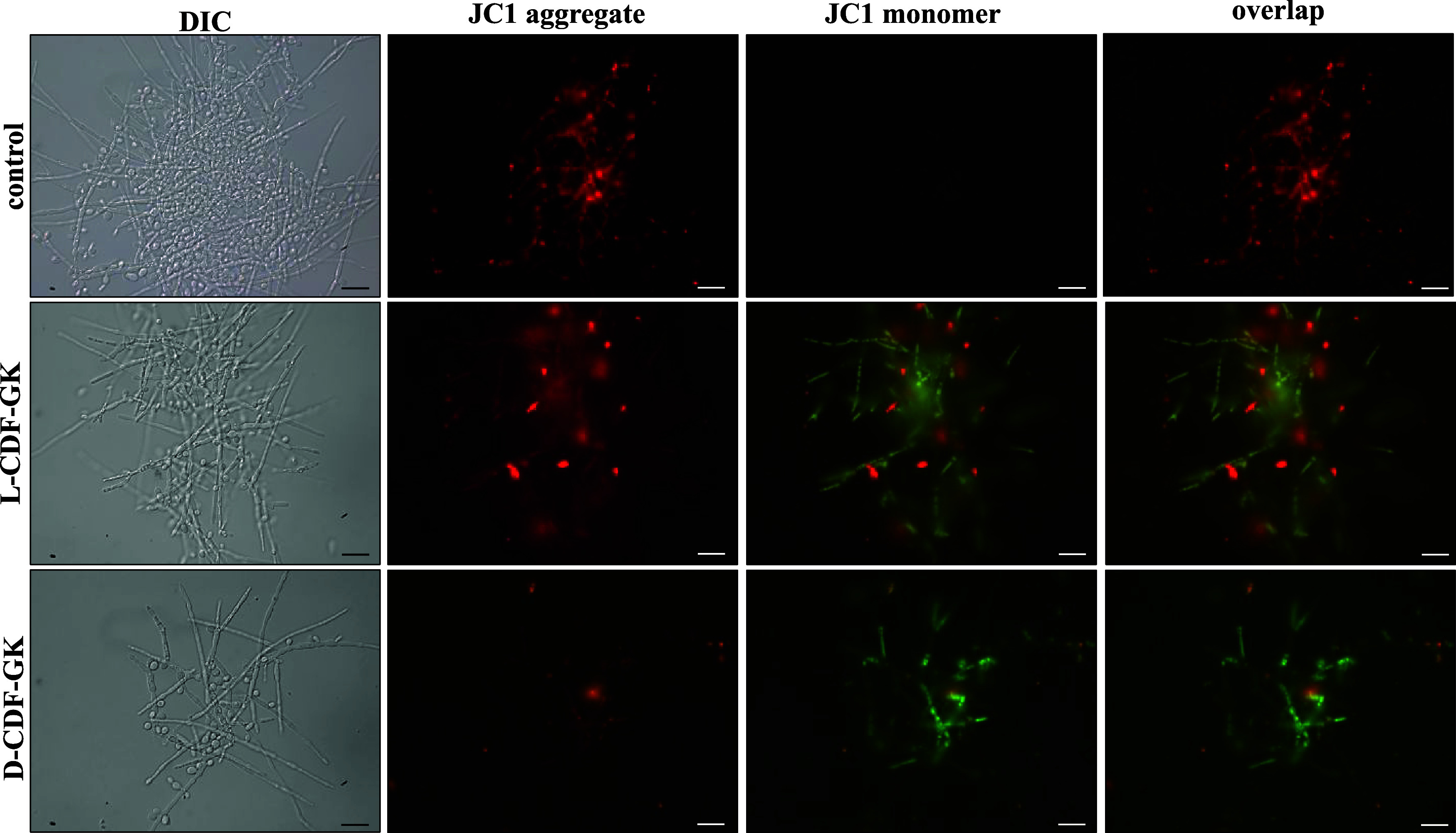
Effect of L-CDF-GK and D-CDF-GK on the mitochondrial membrane potential of *S. schenckii*. Untreated cells (control) and cells treated separately with the IC_50_ of each peptide were stained with JC-1. In cells with preserved mitochondrial membrane potential, JC-1 accumulates as J-aggregates, producing red fluorescence, whereas mitochondrial depolarization prevents aggregate formation and results in green fluorescence from the monomeric form. Scale bars = 20 μm.

### Hemolytic Activity

Hemolytic activity was low for both peptides at all tested concentrations (Figure 5). D-CDF-GK showed significantly lower hemolysis than L-CDF-GK at 25 μM and did not differ from the PBS control. At higher concentrations, neither peptide exceeded 20% hemolysis (Figure 5A). Representative well images showed visible erythrocyte pellet formation in the PBS control and peptide-treated groups, including the highest concentrations tested, whereas Triton X-100 treatment resulted in extensive hemoglobin release into the supernatant (Figure 5B). Together, these data indicate low toxicity toward mammalian erythrocytes and suggest a favorable therapeutic window for both peptides.

**Figure 5:**
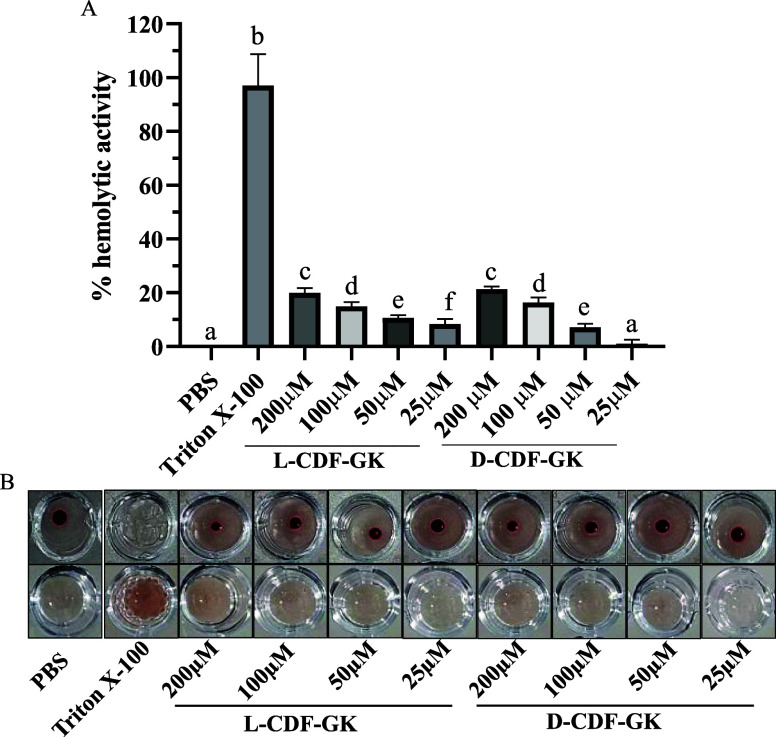
Hemolytic activity of the synthetic peptides L-CDF-GK and D-CDF-GK against erythrocytes. (A) Hemolysis was evaluated after 1 h of incubation at 37 °C with peptide concentrations of 25, 50, 100, and 200 μM. PBS-treated erythrocytes were used as the negative control, whereas erythrocytes treated with 1% (v/v) Triton X-100 were used as the positive control, corresponding to 100% hemolysis. Hemolytic activity is expressed as the percentage of hemolysis relative to the positive and negative controls. Values represent the mean ± SD of triplicates. Different letters indicate statistically significant differences (*p* < 0.05). (B) Representative well images below each condition show the erythrocyte pellet after centrifugation and the corresponding supernatant used for absorbance measurement.

### Toxicity and Effects on Hemocyte Density in *G. mellonella*

The *in vivo* toxicity of the peptides was evaluated in noninfected *G. mellonella* larvae treated with 500 μM/larva of L-CDF-GK or D-CDF-GK and monitored for 7 days (Figure 6A). Survival curves showed that neither peptide caused significant mortality compared with the PBS control, indicating low toxicity at this dose. Likewise, no mortality was observed in the group subjected only to mechanical needle injury. In contrast, the DMSO-treated group showed 100% mortality within 2 days, confirming the deleterious effect of this solvent under the tested conditions.

**Figure 6:**
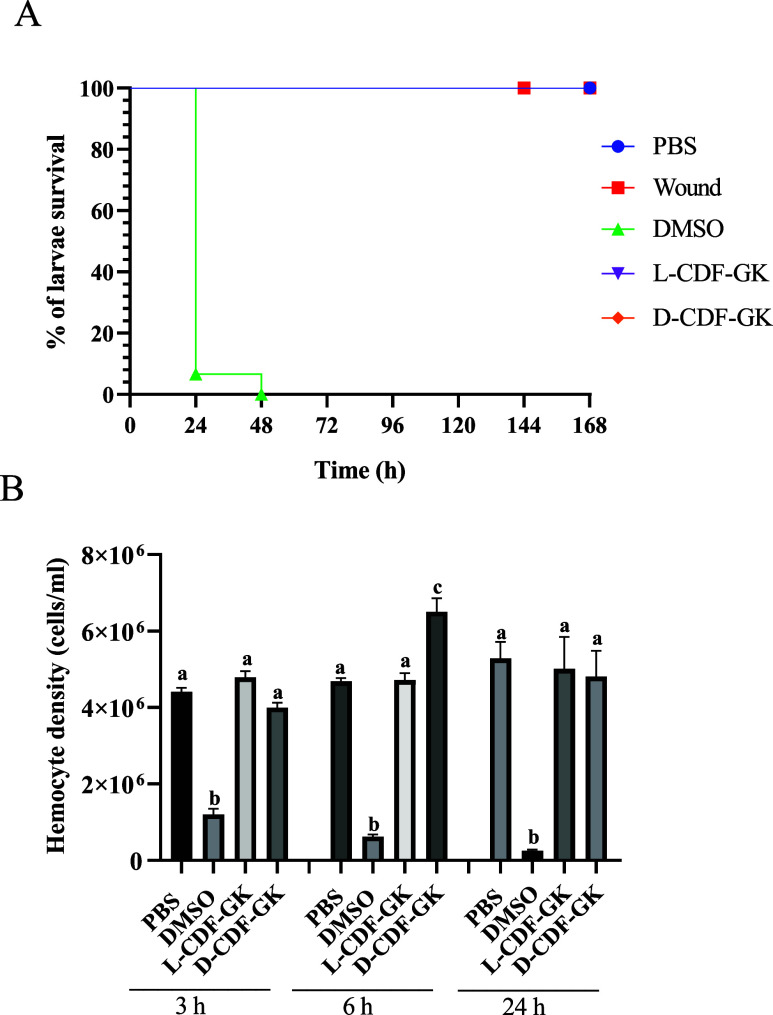
Toxicity of L-CDF-GK and D-CDF-GK in *G. mellonella* larvae. (A) Survival of larvae treated with the peptides, PBS, or DMSO and maintained at 37 °C for 7 days. Results represent the mean of three independent experiments. Statistical significance was determined using the Gehan-Breslow-Wilcoxon test (*p* ≤ 0.05). (B) Hemocyte density in the hemolymph of *G. mellonella* larvae at 3, 6, and 24 h after injection with 500 μM L-CDF-GK, D-CDF-GK, PBS, or DMSO. All larvae were incubated at 37 °C, and hemocyte counts were determined using a hemocytometer. Different letters indicate significant differences, whereas the same letter indicates no significant difference (*p* < 0.05). The experiment was performed in duplicate.

The hemocyte density was analyzed in parallel in the hemolymph of treated larvae (Figure 6B). Both peptides maintained hemocyte counts similar to those of the PBS control, suggesting the absence of cytotoxic effects on these immunocompetent cells. Treatment with D-CDF-GK induced a significant but transient increase in hemocyte density 6 h after injection, with values returning to basal levels at 24 h. In contrast, DMSO reduced hemocyte counts by approximately 50%, consistent with the high mortality observed in this group. Together, these results indicate that L-CDF-GK and D-CDF-GK exhibit low systemic toxicity and do not cause sustained hemocyte depletion in *G. mellonella*.

### *In Vivo* Therapeutic Activity against *S. schenckii* in *G. mellonella*

Pilot virulence assays in *G. mellonella* comparing *S. schenckii* and *S. brasiliensis* are presented in Figure S1. Under the conditions tested, these assays confirmed the greater virulence of *S. brasiliensis*. On the basis of the experimental design adopted in this study, the *in vivo* therapeutic assay was performed using the *S. schenckii* infection model.

The therapeutic potential of the peptides was evaluated in *G. mellonella* larvae infected with 10^6^ cells/larva of *S. schenckii* and treated with L-CDF-GK or D-CDF-GK at 150 or 300 μM. Itraconazole (ITZ, 150 μM) was used as the positive control (Figure 7). Treatment with L-CDF-GK significantly increased larval survival, prolonging viability until day 15, with survival rates of approximately 60% at 300 μM and 30% at 150 μM (Figure 7A). Similarly, D-CDF-GK conferred protection of about 50 and 20% at 300 and 150 μM, respectively (Figure 7B). Although ITZ produced the highest survival rate, reaching approximately 70% at 150 μM, both peptides showed a clear protective effect, particularly at the higher concentration, supporting their therapeutic potential in this infection model. Representative images of the experimental groups on day 15 of incubation (Figure 7C) further illustrate these findings. Noninfected larvae in the PBS group displayed a cream-colored appearance and no visible melanization, whereas infected and untreated larvae were dead and intensely melanized, consistent with disease progression. In contrast, infected larvae treated with the peptides or ITZ showed varying degrees of melanization, in agreement with the survival profiles observed.

**Figure 7:**
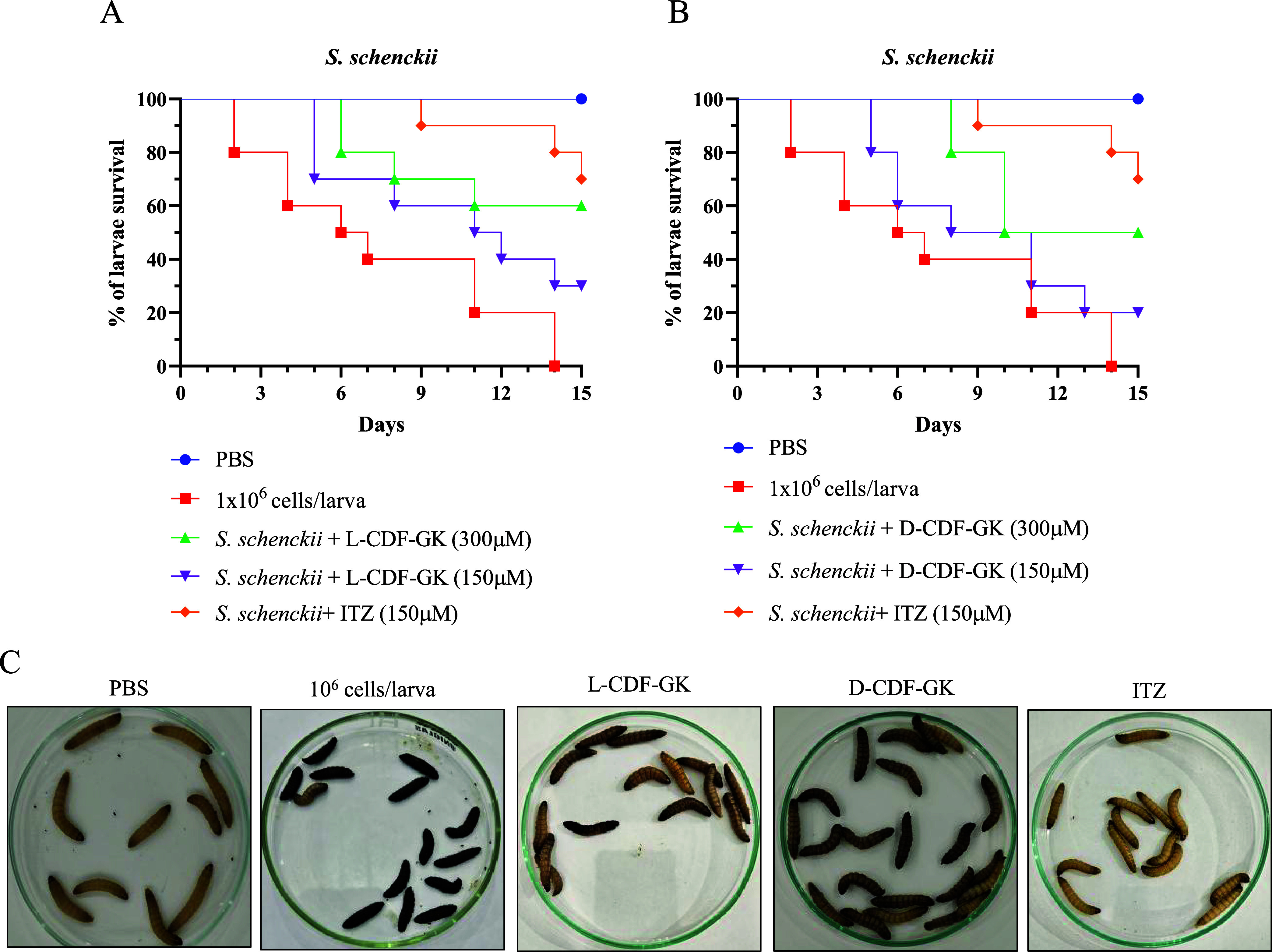
Therapeutic activity of L-CDF-GK and D-CDF-GK in *G. mellonella* larvae infected with *S. schenckii* (10^6^ cells/larva). Survival curves of infected larvae treated with (A) L-CDF-GK or (B) D-CDF-GK at 150 or 300 μM, or with ITZ (150 μM). PBS indicates larvae injected only with phosphate-buffered saline, whereas 10^6^ cells/larva indicates infected, untreated larvae. Results represent the mean of two independent experiments. Statistical significance was determined using the Gehan-Breslow-Wilcoxon test (*p* ≤ 0.05). (C) Representative images of the different larval groups on day 15 of incubation.

### Hemocyte Density during *S. schenckii* Infection and Peptide Treatment

To investigate the larval immune response during *S. schenckii* infection and peptide treatment, hemocyte density was determined in the hemolymph after inoculation with 10^6^ cells/larva and treatment with 300 μM L-CDF-GK or D-CDF-GK (Figure 8). Three main groups were considered: larvae injected only with PBS as the negative control, infected larvae treated with PBS, and infected larvae treated with each peptide. Samples were collected at 3 and 24 h after infection. *S. schenckii* infection caused a significant reduction in hemocyte density relative to the PBS control, particularly at 24 h postinfection, indicating an adverse effect of infection on the innate immune response. In contrast, larvae treated with L-CDF-GK or D-CDF-GK showed a significant recovery in hemocyte counts over time, with higher values at 24 h than those observed in the infected untreated group (Figure 8A). Representative hemolymph images (Figure 8B) showed a predominance of plasmatocytes and granulocytes, key cells of the *G. mellonella* immune system, in the peptide-treated groups, along with fewer yeast-like cells than in the infected group treated only with PBS. Together, these findings suggest that the peptides contribute both to infection control and to the maintenance or restoration of hemocyte density during the infectious process.

**Figure 8:**
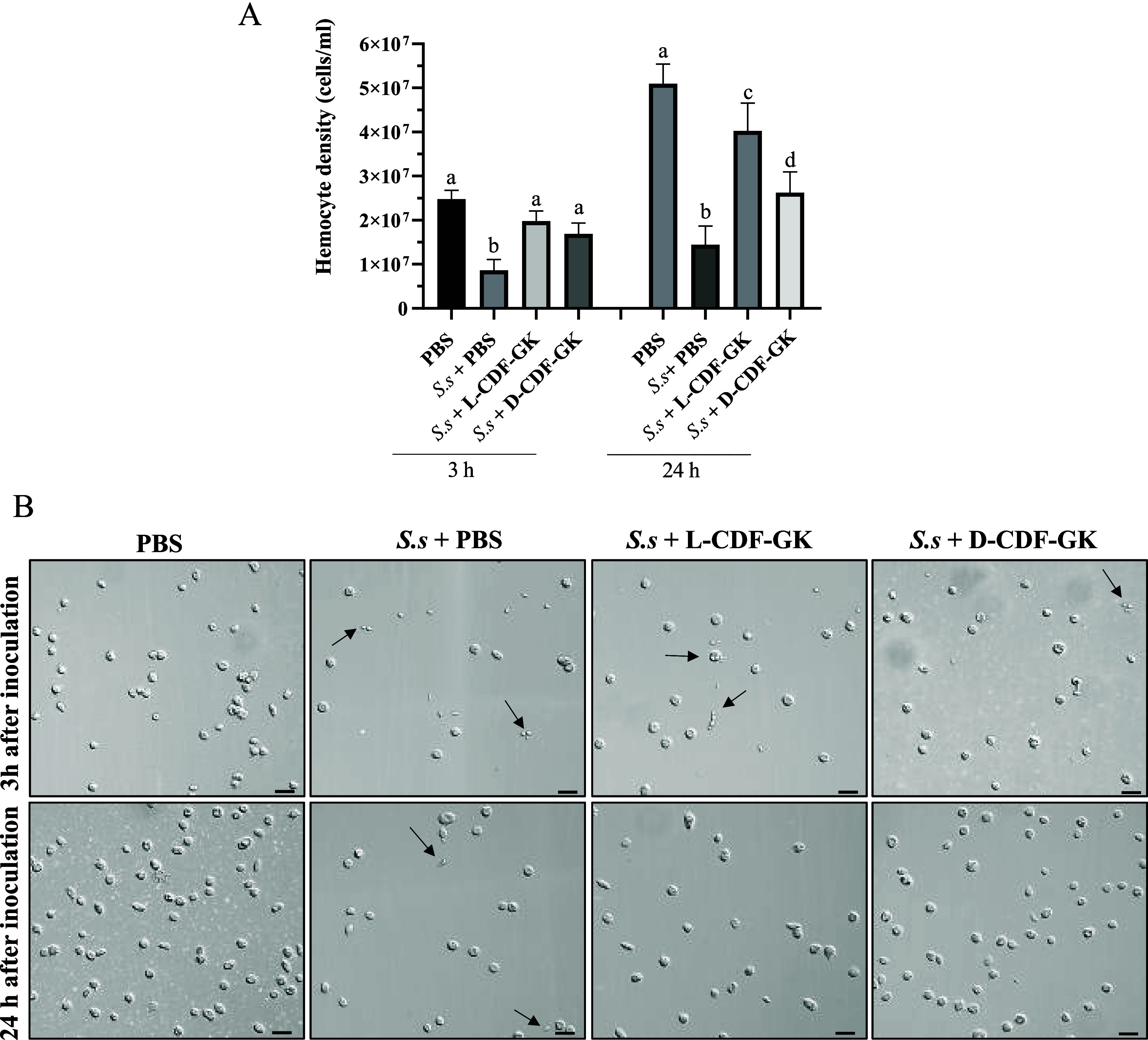
Hemocyte density in *G. mellonella* larvae infected with *S. schenckii*. (A) Hemocyte density in hemolymph collected at 3 and 24 h after infection with 10^6^ cells/larva of *S. schenckii*. Experimental groups included larvae injected with phosphate-buffered saline as the negative control (PBS), infected larvae treated with PBS (*S.s* + PBS), and infected larvae treated with 300 μM D-CDF-GK or L-CDF-GK. Different letters indicate statistically significant differences among groups, whereas identical letters indicate no significant difference (*p* < 0.05). (B) Representative hemolymph images showing the predominance of plasmatocytes and granulocytes, the main phagocytic cells (arrow), at 3 and 24 h postinfection. A higher number of yeast-like cells was observed in the infected untreated group than in the peptide-treated groups, consistent with a lower fungal burden after treatment.

### Melanization during *S. schenckii* Infection and Peptide Treatment

Melanization was evaluated as an additional marker of the *G. mellonella* immune response to *S. schenckii* infection and peptide treatment (Figure 9). The infected group treated only with PBS showed the highest absorbance values, indicating strong activation of the prophenoloxidase cascade and increased melanin deposition, consistent with uncontrolled infection. In contrast, larvae treated with L-CDF-GK or D-CDF-GK exhibited significantly lower absorbance values, suggesting an attenuation of excessive melanization. Among the two peptides, L-CDF-GK promoted the greatest reduction in melanization, in agreement with its superior protective effect in the survival assay and its association with the recovery of hemocyte density. Representative larval images (Figure 9B) support these findings. Infected and untreated larvae displayed intense melanization, particularly along the dorsal vessel, whereas peptide-treated groups showed a lighter coloration, more similar to that of noninfected control larvae. Images of the wells used for hemolymph absorbance measurements likewise revealed greater melanin deposition in the infected untreated group than in the treated groups, reinforcing the association among peptide treatment, reduced melanization, and improved infection outcome.

**Figure 9:**
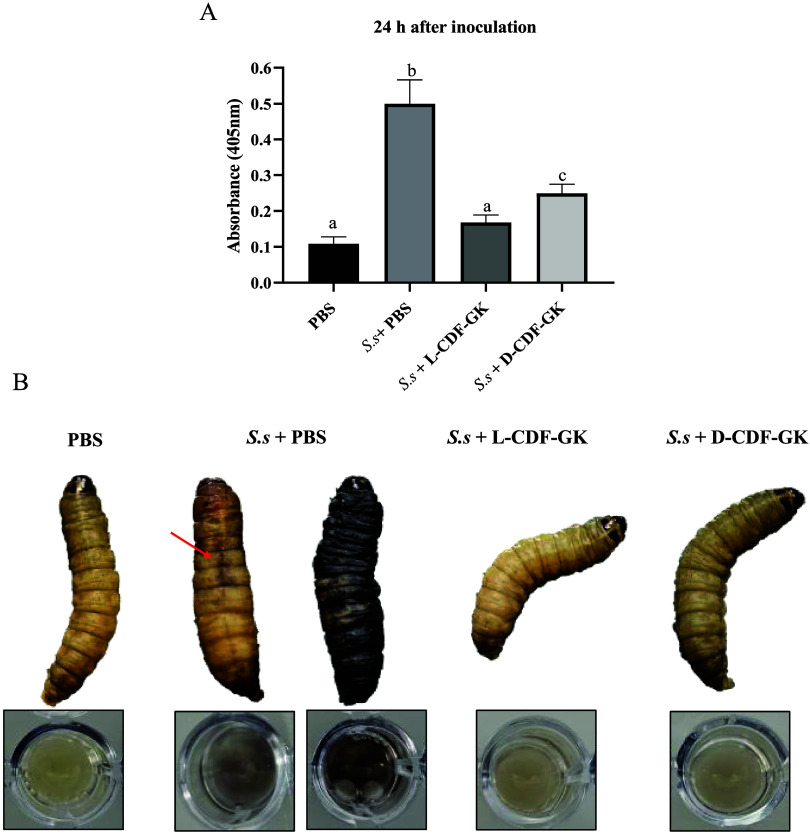
Melanization in *G. mellonella* larvae infected with *S. schenckii*. (A) Optical density of hemolymph collected 24 h after infection with 10^6^ cells/larva of *S. schenckii*. Experimental groups included larvae injected with PBS as the negative control, infected larvae treated with PBS (*S.s* + PBS), and infected larvae treated with 300 μM D-CDF-GK or L-CDF-GK. Different letters indicate statistically significant differences among groups, whereas identical letters indicate no significant difference (*p* < 0.05). (B) Representative images of larvae 24 h after infection, highlighting prominent melanization along the dorsal vessel (arrow) in the infected untreated group compared with peptide-treated groups. Images of the wells used for hemolymph absorbance measurements are also shown.

## Discussion

Sporotrichosis remains a relevant public and veterinary health problem in Brazil, reinforcing the need for new antifungal agents with improved efficacy and low toxicity.^15,16^ In this context, synthetic antimicrobial peptides inspired by natural defense molecules have emerged as promising candidates, particularly when rational design is used to optimize physicochemical properties such as charge, hydrophobicity, and structural stability, which are closely associated with antifungal activity and selectivity.^32,46,47^

CDF-GK is a bioinspired peptide derived from the plant defensin *Ca*Def2.1, originally identified in *C. annuum* and later optimized to increase cationicity and hydrophobicity, two features closely related to membrane interaction and antimicrobial potency.^35,36^ In addition, the synthesis of the d-enantiomer was proposed as a strategy to improve resistance to proteolytic degradation while preserving biological activity, an approach that has been successfully applied to other synthetic AMPs.^48,49^ Previous work from our group showed that L-CDF-GK displays potent antifungal activity against clinically relevant *Candida* species, supporting the evaluation of this scaffold against other pathogenic fungi.^37^

In the present study, both L-CDF-GK and D-CDF-GK exhibited significant dose-dependent antifungal activity against *S. schenckii* and *S. brasiliensis* (Figure 1). Complete growth inhibition at 100 μM was observed for L-CDF-GK, whereas D-CDF-GK also showed inhibitory activity, particularly against *S. schenckii*. To determine whether the complete growth inhibition observed at 100 μM reflected a reversible or irreversible effect, fungal viability was assessed after exposure to L-CDF-GK. After treatment, cells were transferred to peptide-free BHI agar to evaluate colony formation. The absence of colony growth for both *S. schenckii* and *S. brasiliensis* indicates that L-CDF-GK at 100 μM rendered fungal cells nonviable and exerted a fungicidal effect under the tested conditions. Thus, the concentration that completely inhibited fungal growth in the broth microdilution assay also abolished colony-forming capacity, providing additional evidence of the fungicidal profile of L-CDF-GK against *Sporothrix* spp. IC_50_ analysis further supported these findings, revealing greater activity of D-CDF-GK against *S. schenckii* than against *S. brasiliensis*, while L-CDF-GK displayed similar IC_50_ values for both species (Figure 1C). These findings indicate that both enantiomers retain antifungal activity against *Sporothrix* spp., although their effects vary according to species and peptide configuration.

The greater susceptibility observed for *S. schenckii*, particularly to D-CDF-GK, may be partially associated with species-specific differences in cell wall composition and architecture. The cell wall of *Sporothrix* spp. is mainly composed of β-glucans, chitin, and rhamnomannan, and comparative analyses have shown that these components vary between *S. schenckii* and *S. brasiliensis* strains. In particular, *S. brasiliensis* strains have been reported to exhibit higher relative contents of chitin and rhamnomannan than *S. schenckii* strains.^50^ These differences may influence the interaction of cationic amphipathic peptides with the fungal surface by altering cell wall architecture, porosity, and the exposure of surface components, potentially limiting peptide access to the plasma membrane. Therefore, the lower susceptibility of *S. brasiliensis*, particularly to D-CDF-GK, may be partially related to its cell wall polysaccharide composition. However, because cell wall composition was not directly assessed in the present study, further analyses are required to determine how these structural features influence the mechanism of action of CDF-GK-derived peptides against *Sporothrix* spp.

Comparison with the synthetic peptide RP557 further supports the antifungal potential of rationally designed AMPs against clinically relevant fungi. RP557 showed inhibitory activity against most *Sporothrix* isolates in the low-to-intermediate micromolar range and displayed a beneficial interaction with ITZ, although a subset of *S. brasiliensis* isolates remained nonsusceptible.^51^ In parallel, previous studies demonstrated that RP557 also combines membrane-active antifungal activity, limited mammalian toxicity, antibiofilm effects, and *in vivo* efficacy in candidiasis models.^52^ In this context, our findings with L-CDF-GK and D-CDF-GK reinforce the therapeutic value of synthetic AMPs and extend this field by providing mechanistic evidence together with *in vivo* efficacy data in a *G. mellonella* model of *Sporothrix* infection.

A large body of evidence indicates that membrane interactions are a central event in the activity of many antimicrobial peptides. Consistent with this model, both L-CDF-GK and D-CDF-GK were able to compromise the plasma membrane integrity of *S. schenckii* and *S. brasiliensis*, as demonstrated by the Sytox Green assay, with a visually stronger fluorescent signal observed in *S. schenckii* (Figure 2). Because Sytox Green penetrates only cells with damaged membranes and binds intracellular nucleic acids, its uptake provides direct evidence of membrane permeabilization.^53^ The focal fluorescence observed in some peptide-treated cells is consistent with the staining mechanism of Sytox Green, which binds to nucleic acids after entering permeabilized cells. Thus, this focal signal reflects the staining of nuclei. This finding agrees with the classical mode of action proposed for cationic and amphipathic AMPs, which initially interacts with negatively charged microbial membranes through electrostatic attraction, followed by membrane destabilization and pore formation.^54–56^

Our results are also consistent with previous observations obtained with the same peptide against *Candida krusei*, in which membrane permeabilization was likewise demonstrated by Sytox Green staining.^37^ A similar behavior has been reported for other plant-derived or plant-inspired antifungal peptides. For example, the peptide JcTI-PepI, designed from a *Jatropha curcas* trypsin inhibitor, induced membrane permeabilization and subsequent ROS overproduction in *C. krusei*.^57^ Together, these studies support the view that membrane disruption is a recurrent and functionally relevant mechanism among bioinspired antifungal peptides.

In the case of CDF-GK, previous evidence indicates that interaction with fungal membranes rich in ergosterol is associated with peptide rearrangement into an α-helical conformation, a structural transition that may favor lipid destabilization and membrane permeabilization.^37^ Therefore, the permeabilization observed here in *Sporothrix* spp. likely represents an early and conserved event in the antifungal activity of this peptide scaffold, extending a mechanism previously observed in *Candida* to a neglected subcutaneous pathogen. In this context, the more intense fluorescence detected in *S. schenckii* is in line with its greater susceptibility to both peptides in the growth inhibition assays. Moreover, because membrane disruption is often accompanied by intracellular stress responses, these findings support membrane permeabilization as an early event in the mechanism of action of the peptides and provide a basis for the subsequent increase in ROS after treatment.

Consistent with membrane permeabilization as an early event in the mechanism of action of the peptides, fungal cell death is unlikely to be explained by plasma-membrane damage alone. Indeed, analysis of endogenous ROS production using the H_2_DCFDA probe revealed a marked increase in intracellular ROS levels in both *S. schenckii* and *S. brasiliensis* after treatment with L-CDF-GK and D-CDF-GK (Figure 3), again with a stronger signal in *S. schenckii*. Although ROS are physiologically generated in different cell types, with mitochondria representing a major intracellular source, excessive ROS accumulation disrupts redox homeostasis and promotes oxidative damage to proteins, lipids, and DNA.^58–60^ In parallel, the JC-1 assay showed that both peptides induced mitochondrial membrane depolarization in *S. schenckii* (Figure 4), as indicated by reduced red J-aggregate fluorescence and increased green fluorescence. Together, these findings suggest that L-CDF-GK and D-CDF-GK not only disrupt the plasma membrane but also impair mitochondrial function, likely contributing to an oxidative imbalance and loss of fungal viability.^61^

A similar pattern has been reported for other rationally designed antifungal peptides active against *Sporothrix*. Yan et al.^62^ showed that ToAP2D, a peptide inspired by scorpion toxins, exhibited strong antifungal activity against *S. globosa* and induced both ROS accumulation and reduction of Δψm. In that study, these alterations were associated with apoptotic cell death, supported by increased caspase-3-related expression and metacaspase activation. Although apoptotic markers were not evaluated in the present work, the combined occurrence of membrane permeabilization, ROS accumulation, and mitochondrial depolarization supports the view that disruption of redox homeostasis and mitochondrial function is an important component of the mechanism of action of CDF-GK-derived peptides. Membrane damage may facilitate peptide access to intracellular targets or promote ionic imbalance, thereby intensifying ROS generation and amplifying cellular damage.

While these findings support membrane disruption, oxidative imbalance, and mitochondrial dysfunction as important components of the mechanism of action of CDF-GK-derived peptides, their therapeutic potential also depends on safety toward host cells. In this regard, both L-CDF-GK and D-CDF-GK showed low hemolytic activity, remaining below 20% even at the highest concentration tested, and D-CDF-GK displayed even lower hemolysis than L-CDF-GK at 25 μM (Figure 5). This profile suggests favorable selectivity toward fungal cells, a key property for the development of antifungal peptide candidates with reduced host-cell toxicity.^63,64^ Such selectivity is commonly associated with structural and compositional differences between microbial and host membranes, which favor the interaction of cationic AMPs with negatively charged microbial surfaces while reducing damage to mammalian cells.^34,65^

The low hemotoxicity observed here is consistent with the rational design of plant-inspired AMPs and supports the safety of the CDF-GK. In the context of AMP development, the combination of clear antifungal activity with limited hemolytic effects is generally considered to be a favorable indicator of peptide selectivity and therapeutic potential. In this study, both enantiomers maintained low hemolytic activity across the tested concentration range, reinforcing their therapeutic relevance and suggesting that the structural modifications introduced into the peptide sequence were effective not only in preserving antifungal activity but also in limiting toxicity toward mammalian erythrocytes.

This favorable safety profile was further supported by the *in vivo* results obtained in *G. mellonella*. Administration of 500 μM of either peptide did not cause significant larval mortality over 7 days, in clear contrast to DMSO, which was highly toxic under the same conditions (Figure 6A). In addition, hemocyte density remained stable or showed only a transient increase after peptide treatment, returning to basal levels within 24 h, whereas DMSO reduced hemocyte counts by approximately 50% (Figure 6B). Together, these data indicate that L-CDF-GK and D-CDF-GK do not cause relevant systemic toxicity in this model and do not induce sustained depletion of immunocompetent cells, thereby strengthening their candidacy for *in vivo* antifungal application.

Evaluation of *in vivo* toxicity is a critical step in the development of new peptide-based therapeutics, and *G. mellonella* has emerged as a valuable preclinical model because of its practical advantages and the functional relevance of its innate immune system for infection and toxicity studies.^66–68^ Against this background, the safety profile observed for L-CDF-GK and D-CDF-GK is consistent with their further evaluation as antifungal candidates. A related study by Cherene et al.,^64^ which evaluated peptides isolated from *C. annuum* leaves, also showed that toxicity in *G. mellonella* can vary markedly according to peptide sequence and structural class. In that work, the defensin-like peptide CaCDef-like showed no detectable larval toxicity, whereas CaCLTP2 caused only minor mortality and CaCPin-II displayed substantially greater toxicity. The low toxicity of L-CDF-GK and D-CDF-GK, together with the favorable profile of CaCDef-like, supports the view that defensin-inspired peptides may offer enhanced selectivity and safety, although these properties remain strongly sequence-dependent. A broader comparison with other rationally designed peptides further highlights the favorable safety profile of L-CDF-GK and D-CDF-GK. Rocha et al.^69^ reported that the synthetic peptide KW18, although well tolerated in *G. mellonella* and not associated with larval mortality at 4 or 40 μM over 72 h, was still classified as moderately hemolytic, with an HC_50_ of 79.56 μM. In contrast, the low hemolytic activity observed here, together with the absence of relevant toxicity in *G. mellonella*, reinforces the view that the CDF-GK combines antifungal activity with a particularly favorable safety profile. Although direct comparisons between studies should be interpreted cautiously because of differences in peptide purpose, concentration range, and experimental design, these data support the importance of sequence-dependent optimization in improving peptide selectivity and tolerability.

The absence of significant toxicity in *G. mellonella*, together with the preservation of hemocyte homeostasis, supported further evaluation of peptide efficacy *in vivo*. In this regard, *G. mellonella* represents a useful model for antifungal studies, and previous work with *Sporothrix* has shown that it can reproduce virulence trends observed in mammalian systems, including the distinction between *S. schenckii* and *S. brasiliensis*.^70^ In the present study, treatment of *S. schenckii*-infected larvae with L-CDF-GK or D-CDF-GK significantly improved survival in a concentration-dependent manner, indicating that the antifungal activity observed *in vitro* was maintained *in vivo* (Figure 7). Although ITZ produced the highest survival rate, both peptides conferred a clear protective effect, particularly at higher concentrations, supporting their therapeutic relevance in this infection model. These findings are also consistent with previous results obtained with L-CDF-GK against *C. krusei* in *G. mellonella*, in which peptide treatment significantly improved larval survival during lethal infection.^37^ Together, these observations reinforce the broad antifungal potential of this defensin-inspired scaffold in distinct infection settings.

This result is also consistent with the growing evidence that rationally designed AMPs can remain effective *in vivo* against pathogenic fungi. Yan et al.^62^ showed that the synthetic peptide ToAP2D displayed antifungal activity against *S. globosa* and achieved *in vivo* efficacy in a murine footpad infection model comparable to ITZ. Although that study used a different host and fungal species, it similarly supports the feasibility of AMP-based therapeutic strategies against sporotrichosis. In this context, the protective effects of L-CDF-GK and D-CDF-GK in *G. mellonella* expand the evidence supporting defensin-inspired peptides as candidates for antifungal development.

Beyond survival, peptide treatment was associated with modulation of host immune parameters. Infection with *S. schenckii* reduced hemocyte density, whereas treatment with either peptide promoted recovery of circulating hemocyte counts, particularly at 24 h postinfection (Figure 8). A similar trend was previously reported for L-CDF-GK in *C. krusei-*infected larvae, in which peptide treatment restored hemocyte density to levels comparable to those of noninfected controls.^37^ This pattern is also biologically relevant in light of previous observations in the *Sporothrix–Galleria* model. Reis et al.^71^ reported that *S. brasiliensis* infection induced dynamic changes in both cellular and humoral immunity in *G. mellonella*, including variation in hemocyte density together with time-dependent regulation of genes encoding gallerimycin and stress-related proteins. In that study, these changes occurred in parallel with increasing fungal burden and larval mortality, indicating that immune activation alone may not be sufficient to control progressive infection. Although the fungal species and experimental design differ from those used here, these findings strengthen the interpretation that restoration of hemocyte density in peptide-treated larvae is consistent with improved infection control rather than simply nonspecific immune activation.

A similar rationale applies to the melanization. In the present study, infected untreated larvae showed the highest melanization levels, whereas treatment with L-CDF-GK or D-CDF-GK reduced this response, with the l enantiomer showing the strongest effect (Figure 9). Because melanization is a major humoral immune process in insects, initiated by activation of the prophenoloxidase cascade, its attenuation in treated larvae is more consistent with reduced infection-associated immune stress than with worsening disease.^72,73^ This interpretation is in agreement with previous observations obtained with L-CDF-GK in the *C. krusei* model, in which peptide treatment also reduced melanization while increasing larval survival,^37^ and is further supported by direct evidence that melanization in *G. mellonella* functions as an antifungal defense mechanism *in vivo*.^74^ Taken together, the recovery of hemocyte density and the reduction in melanization indicate that peptide treatment not only prolongs survival but is also associated with a more favorable host response profile during infection.

Overall, the *in vivo* assays indicate that L-CDF-GK and D-CDF-GK display a favorable therapeutic profile in *G. mellonella* infected with *S. schenckii*, reflected by increased survival together with modulation of relevant immune markers. These findings complement the *in vitro* evidence of membrane permeabilization, ROS accumulation, and changes in the mitochondrial membrane potential, supporting a multifactorial mechanism of action combined with low host toxicity. A limitation of the present study is that the *in vivo* therapeutic evaluation was performed only in the *S. schenckii* infection model. Given the greater virulence of *S. brasiliensis* in *G. mellonella*, both in our pilot assays and in previous reports, future studies should determine whether the protective effects observed here are maintained against this species.^70^

From a translational perspective, the choice of an appropriate vehicle will be an important step in the future development of CDF-GK-derived peptides. In the present study, peptide administration in *G. mellonella* was used as a proof-of-concept approach to evaluate toxicity and therapeutic potential. For future applications, vehicle selection should be guided by the intended route of administration and clinical presentation of sporotrichosis. Considering that sporotrichosis commonly presents as cutaneous or subcutaneous lesions, topical or local delivery systems, such as hydrogels, creams, or wound-dressing formulations, may be useful to improve peptide retention at the infection site and limit systemic exposure. In contrast, systemic or disseminated forms would require sterile aqueous formulations suitable for parenteral administration with additional evaluation of peptide solubility, stability, pharmacokinetics, and safety. Therefore, formulation studies will be necessary to define the most appropriate vehicle for the administration of L-CDF-GK and D-CDF-GK.^75–77^

Even with the current limitations, the present results support CDF-GK as a promising defensin-inspired scaffold for the development of new antifungal strategies against sporotrichosis.

## Conclusion

In conclusion, L-CDF-GK and D-CDF-GK showed relevant antifungal activity against pathogenic *Sporothrix* species and were associated with low toxicity in both *in vitro* and *in vivo* models. The results support CDF-GK as a promising defensin-inspired scaffold and expand the current understanding of synthetic peptide candidates for sporotrichosis. Future studies should assess a broader panel of clinical isolates, determine whether the protective effects observed in *G. mellonella* are maintained against *S. brasiliensis*, and further validate these peptides in more complex experimental models.

## Supplementary Material



## Notes

Survival curves used to determine the lethal fungal dose of *S. schenckii* and *S. brasiliensis* in *G. mellonella* larvae (PDF)

## Abbreviations

L-CDF-GK: L-CaDef2.1G27-K44
D-CDF-GK: D-CaDef2.1G27-K44
ITZ: itraconazole

## References

[1] Vollset S. E., Ikuta K. S., GBD
2021 Antimicrobial Resistance Collaborators (2024). Global
Burden of Bacterial Antimicrobial Resistance 1990–2021: A Systematic
Analysis with Forecasts to 2050. Lancet.

[2] WHO - World Health Organization . Fungal priority pathogens list to guide research, development and public health action, https://www.who.int/publications/i/item/9789240060241 (accessed Jan, 2026).

[3] Brown G. D., Ballou E. R., Bates S., Bignell E. M., Borman A. M., Brand A. C., Brown A. J. P., Coelho C., Cook P. C., Farrer R. A., Govender N. P., Gow N. A. R., Hope W., Hoving J. C., Dangarembizi R., Harrison T. S., Johnson E. M., Mukaremera L., Ramsdale M., Thornton C. R., Usher J., Warris A., Wilson D. (2024). The Pathobiology of Human Fungal
Infections. Nat. Rev. Microbiol..

[4] Denning D. W. (2024). Global
Incidence and Mortality of Severe Fungal Disease—Author’s
Reply. Lancet Infect. Dis..

[5] Shah K., Deshpande M., Shah P. (2024). Healthcare-Associated Fungal Infections
and Emerging Pathogens during the COVID-19 Pandemic. Front. Fungal Biol..

[6] Giacomazzi J., Baethgen L., Carneiro L. C., Millington M. A., Denning D. W., Colombo A. L., Pasqualotto A. C., Association With The LIFE Program (2016). The Burden of Serious Human Fungal Infections in Brazil. Mycoses.

[7] Prohaska-Batista F., Bridi Cavassin F., de Queiroz-Telles F. (2025). From Neglected to Notable: A Growing
Public Health Challenge Driven by Hospitalization for Sporotrichosis
in Pernambuco, Northeast Brazil (2016–2024). PLoS Neglected Trop. Dis.

[8] Ribeiro
dos Santos A., Misas E., Min B., Le N., Bagal U. R., Parnell L. A., Sexton D. J., Lockhart S. R., de Souza Carvalho Melhem M., Takahashi J. P. F., Oliboni G. M., Bonfieti L. X., Cappellano P., Sampaio J. L. M., Araujo L. S., Alves Filho H. L., Venturini J., Chiller T. M., Litvintseva A. P., Chow N. A. (2024). Emergence of Zoonotic Sporotrichosis in Brazil: A Genomic
Epidemiology Study. Lancet Microbe.

[9] Poester V. R., Xavier M. O., Munhoz L. S., Basso R. P., Zancopé-Oliveira R. M., Freitas D. F. S., Pasqualotto A. C. (2024). *Sporothrix brasiliensis* Causing Atypical Sporotrichosis in Brazil: A Systematic Review. J. Fungi.

[10] Dixon D. M., Salkin I. F., Duncan R. A., Hurd N. J., Haines J. H., Kemna M. E., Coles F. B. (1991). Isolation and Characterization of *Sporothrix schenckii* from Clinical and Environmental
Sources Associated with the Largest U.S. Epidemic of Sporotrichosis. J. Clin. Microbiol..

[11] Barros M. B. d. L., de Almeida Paes R., Schubach A. O. (2011). *Sporothrix
schenckii* and Sporotrichosis. Clin. Microbiol. Rev..

[12] Chakrabarti A., Bonifaz A., Gutierrez-Galhardo M.
C., Mochizuki T., Li S. (2015). Global Epidemiology of Sporotrichosis. Med.
Mycol..

[13] Marimon R., Gené J., Cano J., Trilles L., Dos Santos
Lazéra M., Guarro J. (2006). Molecular Phylogeny of *Sporothrix schenckii*. J. Clin.
Microbiol..

[14] Morgado D. S., Castro R., Ribeiro-Alves M., Corrêa-Moreira D., Silva J. C. A. L. E., Menezes R. C., Oliveira M. M. E. (2024). Systematic Review
of Literature to Evaluate Global Distribution of Species of the *Sporothrix* Genus Stored in Culture Collections. Front. Cell. Infect. Microbiol..

[15] Gremião I. D.
F., Miranda L. H., Reis E. G., Rodrigues A. M., Pereira S. A. (2017). Zoonotic Epidemic
of Sporotrichosis: Cat to Human Transmission. PLoS Pathog..

[16] Gremião I. D. F., Oliveira M. M. E., Monteiro de Miranda L. H., Saraiva Freitas D. F., Pereira S. A. (2020). Geographic Expansion of Sporotrichosis,
Brazil. Emerg. Infect. Dis..

[17] Cognialli R. C. R., Queiroz-Telles F., Cavanaugh A. M., Rediguieri B. C., Santos G. C. D., Matias F. M., Filho L. M., Neto E. N. M., Surek M., Vicente V. A., Santos-Weiss I. C. R. (2025). New
Insights on Transmission of *Sporothrix brasiliensis*. Mycoses.

[18] Gómez-Gaviria M., Martínez-Álvarez J. A., Mora-Montes H. M. (2023). Current
Progress in *Sporothrix brasiliensis* Basic Aspects. J. Fungi.

[19] Matute D. R., Teixeira M. M. (2025). *Sporothrix* Is Neglected among the
Neglected. PLoS Pathog..

[20] Orofino-Costa R., Freitas D. F. S., Bernardes-Engemann A.
R., Rodrigues A. M., Talhari C., Ferraz C. E., Veasey J. V., Quintella L., Sousa M. S. L. A., Vettorato R., Almeida-Paes R., de Macedo P. M. (2022). Human Sporotrichosis: Recommendations from the Brazilian
Society of Dermatology for the Clinical, Diagnostic and Therapeutic
Management. An. Bras. Dermatol..

[21] Freitas D. F. S., Cunha R. P., de Oliveira R. V. C., de Macedo P. M., do Valle A. C. F., Rezende A. P. M. B., Eiras R. V., Curi A. L. L., Carvalho E. M., de Moraes R. L., Almeida-Paes R., Zancopé-Oliveira R. M., Gutierrez-Galhardo M. C. (2024). Sporotrichosis
during Pregnancy: A Retrospective Study of 58 Cases in a Reference
Center from 1998 to 2023. PLoS Neglected Trop.
Dis..

[22] Almeida-Paes R., do Valle A. C. F., Freitas D. F. S., de Macedo P. M., Zancopé-Oliveira R. M., Gutierrez-Galhardo M. C. (2024). The Present
and Future Research Agenda of Sporotrichosis on the Silver Anniversary
of Zoonotic Sporotrichosis in Rio de Janeiro, Brazil. Mem. Inst. Oswaldo Cruz.

[23] Veasey J. V., Reis A. P. C., Celestrino G. A., Silva C. E., Santos E. S., Mendes D. P., Andrade T. S., Bonfietti L. X., Benard G., Sousa M. G. T. (2024). Evidence of Clinical
and Laboratory
Correlation of Itraconazole Resistance in *Sporothrix
brasiliensis* Infection: Case Report. Microorganisms.

[24] do
Prado C. M., Spruijtenburg B., Razzolini E., Chiyo L., Santi C., Martins C. A., Santacruz G., Segovia N., Brunelli J. P., Cognialli R. C. R., Meis J. F., Vicente V. A., de Groot T., Meijer E. F. J., Queiroz-Telles F. (2025). *Sporothrix brasiliensis* Treatment Failure without Initial Elevated Itraconazole MICs in
Felids at Border of Brazil. Emerg. Infect. Dis..

[25] Nakasu C. C. T., Waller S. B., Ripoll M. K., Ferreira M. R. A., Conceição F. R., Gomes A. D. R., Osório L. D. G., de Faria R. O., Cleff M. B. (2021). Feline
Sporotrichosis: A Case Series of Itraconazole-Resistant *Sporothrix brasiliensis* Infection. Braz. J. Microbiol..

[26] Waller S. B., Dalla Lana D. F., Quatrin P. M., Ferreira M. R. A., Fuentefria A. M., Mezzari A. (2021). Antifungal Resistance
on *Sporothrix* Species: An Overview. Braz. J. Microbiol..

[27] Almeida-Paes R., de Oliveira L. C., Oliveira M. M., Gutierrez-Galhardo M. C., Nosanchuk J. D., Zancopé-Oliveira R. M. (2015). Phenotypic Characteristics
Associated with Virulence of Clinical Isolates from the *Sporothrix* Complex. BioMed. Res. Int..

[28] Lopes-Bezerra L. M., Mora-Montes H. M., Zhang Y., Nino-Vega G., Rodrigues A. M., de Camargo Z. P., de Hoog S. (2018). Sporotrichosis between
1898 and 2017: The Evolution of Knowledge on a Changeable Disease
and on Emerging Etiological Agents. Med. Mycol..

[29] Schapiro S., Pulciano N., Galindo-Ramirez J., Rau N., Tuells J., Agudelo Higuita N. I., Thompson G. R., Chastain D. B., Henao-Martínez A. F. (2026). Epidemiology and
Outcomes of Sporotrichosis:
A Descriptive Real-World Analysis From a Global Cohort. Mycoses.

[30] Ma X., Wang Q., Ren K., Xu T., Zhang Z., Xu M., Rao Z., Zhang X. (2024). A Review of Antimicrobial Peptides:
Structure, Mechanism of Action, and Molecular Optimization Strategies. Fermentation.

[31] Marciano C. L., Félix de Lima J. V., Couto
Rosa M. S. D., do Nascimento R. A., Ferraz A. O., Silva I. C. D., Chrysostomo-Massaro T. N., Rosa-Garzon N. G. D., Cabral H. (2025). A Comprehensive Overview of Antimicrobial Peptides:
Broad-Spectrum Activity, Computational Approaches, and Applications. Antibiotics.

[32] Váradi G., Bende G., Borics A., Dán K., Rákhely G., Tóth G. K., Galgóczy L. (2024). Rational Design
of Antifungal Peptides Based on the γ-Core Motif of a *Neosartorya (Aspergillus) fischeri* Antifungal Protein to
Improve Structural Integrity, Efficacy, and Spectrum. ACS Omega.

[33] Sharma A., Singh G., Bhatti J. S., Gill S. K., Arya S. K. (2025). Antifungal
Peptides: Therapeutic Potential and Challenges before Their Commercial
Success. Int. J. Biol. Macromol..

[34] Gong Y., Xue Q., Li J., Zhang S. (2024). Antifungal Peptides from Living Organisms. Front. Microbiol..

[35] Gebara R. S., Taveira G. B., de Azevedo Dos Santos L., Calixto S. D., Simão T. L. B.
V., Lassounskaia E., Muzitano M. F., Teixeira-Ferreira A., Perales J., Rodrigues R., de Oliveira Carvalho A., Gomes V. M. (2020). Identification and Characterization
of Two Defensins from *Capsicum annuum* Fruits That Exhibit Antimicrobial Activity. *Probiotics Antimicrob*. Proteins.

[36] Taveira G. B., de Oliveira Mello É., Simão T. L. B. V., Cherene M. B., de Oliveira Carvalho A., Muzitano M. F., Lassounskaia E., Pireda S., de Castro Miguel E., Basso L. G. M., Da Cunha M., da Motta O. V., Gomes V. M. (2022). A New Bioinspired
Peptide on Defensin from *Capsicum annuum* Fruits: Antimicrobial Activity, Mechanisms of Action and Therapeutical
Potential. Biochim. Biophys. Acta, Gen. Subj..

[37] Guimarães T. Z.
A., Mello É. O., Lucas D. R., Damica F. Z., Magalhães F. S. S., Basso L. G. M., Carvalho A. O., Gomes V. M., Taveira G. B. (2025). In Vitro
and In Vivo Antifungal Efficacy
and Safety of the CaDef2.1G27-K44 Peptide against the Neglected and
Drug-Resistant Pathogen *Candida krusei*. ACS Bio Med. Chem. Au.

[38] Jia F., Wang J., Peng J., Zhao P., Kong Z., Wang K., Yan W., Wang R. (2017). D-Amino Acid Substitution
Enhances the Stability of Antimicrobial Peptide Polybia-CP. Acta Biochim. Biophys. Sin..

[39] Thevissen K., Terras F. R., Broekaert W. F. (1999). Permeabilization
of Fungal Membranes
by Plant Defensins Inhibits Fungal Growth. Appl.
Environ. Microbiol..

[40] Mello E. O., Ribeiro S. F., Carvalho A. O., Santos I. S., Da Cunha M., Santa-Catarina C., Gomes V. M. (2011). Antifungal Activity of PvD1 Defensin
Involves Plasma Membrane Permeabilization, Inhibition of Medium Acidification,
and Induction of ROS in Fungi Cells. Curr. Microbiol..

[41] Qian Y., Kachroo A. H., Yellman C. M., Marcotte E. M., Johnson K. A. (2014). Yeast Cells
Expressing the Human Mitochondrial DNA Polymerase Reveal Correlations
between Polymerase Fidelity and Human Disease Progression. J. Biol. Chem..

[42] Oren Z., Shai Y. (1997). Selective Lysis of Bacteria but Not Mammalian Cells by Diastereomers
of Melittin: Structure-Function Study. Biochemistry.

[43] Jorjão A. L., Oliveira L. D., Scorzoni L., Figueiredo-Godoi L. M. A., Prata M. C. A., Jorge A. O. C., Junqueira J. C. (2018). From Moths
to Caterpillars: Ideal Conditions for *Galleria mellonella* Rearing for In Vivo Microbiological Studies. Virulence.

[44] Mylonakis E., Moreno R., El Khoury J. B., Idnurm A., Heitman J., Calderwood S. B., Ausubel F. M., Diener A. (2005). *Galleria
mellonella* as a Model System to Study *Cryptococcus
neoformans* Pathogenesis. Infect. Immun..

[45] Scorzoni L., de Lucas M. P., Mesa-Arango A. C., Fusco-Almeida A. M., Lozano E., Cuenca-Estrella M., Mendes-Giannini M. J., Zaragoza O. (2013). Antifungal Efficacy during *Candida krusei* Infection in Non-Conventional Models
Correlates with the Yeast In
Vitro Susceptibility Profile. PLoS One.

[46] Xu R., Tang J., Hadianamrei R., Liu S., Lv S., You R., Pan F., Zhang P., Wang N., Cai Z., Zhao X. (2023). Antifungal Activity
of Designed α-Helical Antimicrobial Peptides. Biomater. Sci..

[47] Sadasivam D., Nambiar P., Dutta A., Mitra D. (2024). Rational Design of
Antimicrobial Peptides: An Optimization Approach. Mol. Syst. Des. Eng..

[48] Toledo E. B., Lucas D. R., Simão T. L. B.
V., Calixto S. D., Lassounskaia E., Muzitano M. F., Damica F. Z., Gomes V. M., de Oliveira Carvalho A. (2021). Design of Improved Synthetic Antifungal Peptides with
Targeted Variations in Charge, Hydrophobicity and Chirality Based
on a Correlation Study between Biological Activity and Primary Structure
of Plant Defensin γ-Cores. Amino Acids.

[49] Oliveira
Júnior N. G., Souza C. M., Buccini D. F., Cardoso M. H., Franco O. L. (2025). Antimicrobial Peptides: Structure, Functions and Translational
Applications. Nat. Rev. Microbiol..

[50] Villalobos-Duno H. L., Barreto L. A., Alvarez-Aular Á., Mora-Montes H. M., Lozoya-Pérez N. E., Franco B., Lopes-Bezerra L. M., Niño-Vega G. A. (2021). Comparison
of Cell Wall Polysaccharide Composition
and Structure Between Strains of *Sporothrix schenckii* and *Sporothrix brasiliensis*. Front. Microbiol..

[51] Poester V. R., Xavier M. O., Hidalgo J. E. D., Dos
Santos M. C., Trápaga M. R., Al-Hatmi A. M. S., Jaynes J., Stevens D. A. (2026). Antifungal
Activity of the Antimicrobial Peptide RP557 against Priority Fungal
Pathogens. Microbiology.

[52] Woodburn K. W., Clemens L. E., Jaynes J., Joubert L. M., Botha A., Nazik H., Stevens D. A. (2019). Designed
Antimicrobial Peptides for
Recurrent Vulvovaginal Candidiasis Treatment. Antimicrob. Agents Chemother..

[53] Pereira J. V., Gamage H. K. A. H., Cain A. K., Hayes E., Paulsen I. T., Tetu S. G. (2023). High-Throughput Viability Testing of Microbial Communities
in a Probiotic Product Using Flow Cytometry. Appl. Microbiol..

[54] Koehbach J., Craik D. J. (2019). The Vast Structural Diversity of
Antimicrobial Peptides. Trends Pharmacol. Sci..

[55] Chen N., Jiang C. (2023). Antimicrobial Peptides:
Structure, Mechanism, and Modification. Eur.
J. Med. Chem..

[56] Roque-Borda C. A., Primo L. M. D. G., Medina-Alarcón K. P., Campos I. C., Nascimento C. F., Saraiva M. M. S., Berchieri
Junior A., Fusco-Almeida A. M., Mendes-Giannini M. J.
S., Perdigão J., Pavan F. R., Albericio F. (2025). Antimicrobial Peptides: A Promising
Alternative to Conventional Antimicrobials for Combating Polymicrobial
Biofilms. Adv. Sci..

[57] Souza L. A. L., Dias L. P., Araújo N. M. S., Carneiro R. F., Nagano C. S., Teixeira C. S., Silva R. G. G., Oliveira J. T. A., Sousa D. O. B. (2022). JcTI-PepI,
a Synthetic Peptide Bioinspired in the Trypsin Inhibitor from *Jatropha curcas*, Presents Potent Inhibitory Activity
against *Candida krusei*, a Neglected
Pathogen. Biochimie.

[58] Zhang B., Pan C., Feng C., Yan C., Yu Y., Chen Z., Guo C., Wang X. (2022). Role of Mitochondrial
Reactive Oxygen Species in Homeostasis
Regulation. Redox Rep..

[59] Okoye C. N., Koren S. A., Wojtovich A. P. (2023). Mitochondrial
Complex I ROS Production
and Redox Signaling in Hypoxia. Redox Biol..

[60] Li B., Ming H., Qin S., Nice E. C., Dong J., Du Z., Huang C. (2025). Redox Regulation:
Mechanisms, Biology and Therapeutic
Targets in Diseases. Signal Transduction Targeted
Ther..

[61] Li R., Zhao J., Huang L., Yi Y., Li A., Li D., Tao M., Liu Y. (2020). Antimicrobial
Peptide CGA-N12 Decreases
the *Candida tropicalis* Mitochondrial Membrane Potential
via Mitochondrial Permeability Transition Pore. Biosci. Rep..

[62] Yan T., Li F., Li J., Chen F. (2021). Antifungal Activity of ToAP2D Peptide
Against *Sporothrix globosa*. Front. Bioeng. Biotechnol..

[63] Fernández
de Ullivarri M., Arbulu S., Garcia-Gutierrez E., Cotter P. D. (2020). Antifungal Peptides as Therapeutic Agents. Front. Cell. Infect. Microbiol..

[64] Cherene M. B., Cavaco M. C., Neves V. L. S., de Lima Simões C., Basso L. G. M., Silva J. C. A., Gomes V. M., Taveira G. B. (2024). Non-toxicity of Plant
Candicidal Peptides for Mammalian Cell Lines
and *Galleria mellonella* Model to Improving
Selectivity for Clinical Use. Int. J. Pept.
Res. Ther..

[65] Ngashangva N., Huidrom S., Devi I. S. (2026). Antimicrobial
Peptides: Natural Templates
for Next-Generation Therapeutics against Antimicrobial Resistance. Front. Cell. Infect. Microbiol..

[66] Curtis A., Binder U., Kavanagh K. (2022). *Galleria mellonella* Larvae as a Model for Investigating
Fungal-Host Interactions. Front. Fungal Biol..

[67] Giammarino A., Bellucci N., Angiolella L. (2024). *Galleria mellonella* as a Model for the Study of Fungal
Pathogens: Advantages and Disadvantages. Pathogens.

[68] Marena G. D., Thomaz L., Nosanchuk J. D., Taborda C. P. (2025). *Galleria
mellonella* as an Invertebrate Model for Studying Fungal
Infections. J. Fungi.

[69] Rocha L. S., Jacobowski A. C., Thiburcio E., Pereira R. A., Almeida C. V., Gutierrez C. O., de Andrade Farias Rodrigues T., Oliveira R. J., Taveira G. B., Hiane P. A., de Araújo
Boleti A. P., Franco O. L., Cardoso M. H., Macedo M. L. R. (2026). Rationally
Designed Peptide Induces Apoptosis and Cell Cycle Modulation in Resistant
Melanoma. Biochim. Biophys. Acta, Gen. Subj..

[70] Clavijo-Giraldo D. M., Matínez-Alvarez J. A., Lopes-Bezerra L. M., Ponce-Noyola P., Franco B., Almeida R. S., Mora-Montes H. M. (2016). Analysis
of *Sporothrix schenckii* sensu stricto
and *Sporothrix brasiliensis* Virulence
in *Galleria mellonella*. J. Microbiol. Methods.

[71] Reis N. F., de Jesus M. C. S., de Souza L. C. D. S.
V., Alcântara L. M., Rodrigues J. A. C., Brito S. C. P., Penna P. A., Vieira C. S., Silva J. R. S., Penna B. A., Machado R. L. D., Mora-Montes H. M., Baptista A. R. S. (2023). *Sporothrix brasiliensis* Infection Modulates Antimicrobial Peptides and Stress Management
Gene Expression in the Invertebrate Biomodel *Galleria
mellonella*. J. Fungi.

[72] Wojda I. (2017). Immunity of
the Greater Wax Moth *Galleria mellonella*. Insect Sci..

[73] Zdybicka-Barabas A., Stączek S., Kunat-Budzyńska M., Cytryńska M. (2025). Innate Immunity
in Insects: The Lights and Shadows of Phenoloxidase System Activation. Int. J. Mol. Sci..

[74] Smith D. F. Q., Dragotakes Q., Kulkarni M. (2022). *Galleria
mellonella* Immune Melanization Is Fungicidal during
Infection. Commun. Biol..

[75] Thapa R. K., Diep D. B., Tønnesen H. H. (2020). Topical
Antimicrobial Peptide Formulations
for Wound Healing: Current Developments and Future Prospects. Acta Biomater..

[76] Li G., Lai Z., Shan A. (2023). Advances of Antimicrobial Peptide-Based
Biomaterials
for the Treatment of Bacterial Infections. Adv.
Sci..

[77] Cresti L., Cappello G., Pini A. (2024). Antimicrobial
Peptides towards Clinical
Application—A Long History to Be Concluded. Int. J. Mol. Sci..

