# Supplementary material for: Antifungal and Therapeutic Evaluation of l- and d‑Enantiomers of a Plant Defensin-Inspired Peptide against Sporothrix schenckii and Sporothrix brasiliensis
Source: ACS Omega. 2026 Jun 4;11(23):34738–53. doi: 10.1021/acsomega.6c04139 (PMC13280837; doi:10.1021/acsomega.6c04139)
Supplement: Supplementary file 1 [file ao6c04139_si_001.pdf]

Antifungal and therapeutic evaluation of L- and D-enantiomers of a plant defensin-inspired peptide against *Sporothrix schenckii* and *Sporothrix brasiliensis*

Jeniffer Cristina Serpa da Rosa Santos, Thomas Zacarone Afonso Guimarães, Érica de Oliveira Mello, André de Oliveira Carvalho, Valdirene Moreira Gomes, Gabriel Bonan Taveira*

Laboratório de Fisiologia e Bioquímica de Microrganismos, Centro de Biociências e Biotecnologia, Universidade Estadual do Norte Fluminense Darcy Ribeiro, Campos dos Goytacazes, RJ, postal code: 28013-602, Brazil

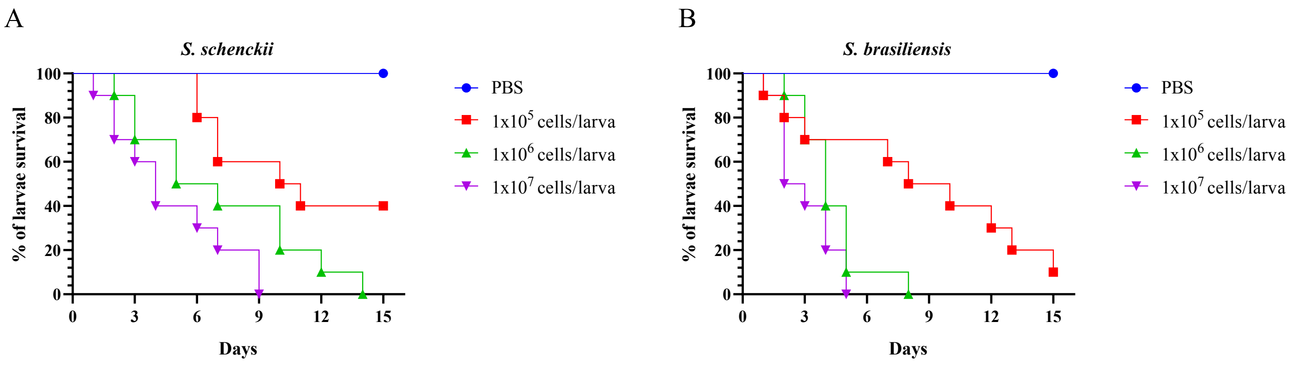

Figure S1. Determination of the lethal fungal dose in *G. mellonella* larvae infected with *S. schenckii* (A) or *S. brasiliensis* (B). Survival curves were generated using inocula ranging from 10^5 to 10^7 cells/larva. Larvae injected with PBS alone were used as the control group. Results represent the mean of two independent experiments. Statistical significance was determined using the Gehan-Breslow-Wilcoxon test ($p \leq 0.05$).
